# Lipid Bilayer Strengthens the Cooperative Network of a Membrane-Integral Enzyme

**DOI:** 10.1101/2023.05.30.542905

**Published:** 2023-05-31

**Authors:** Shaima Muhammednazaar, Jiaqi Yao, Ruiqiong Guo, May S. Rhee, Kelly H. Kim, Seung-gu Kang, Heedeok Hong

**Affiliations:** 1 Department of Chemistry, Michigan State University, East Lansing, MI 48824, USA; 2 Department of Biochemistry & Molecular Biology, Michigan State University, East Lansing, MI 48824, USA; 3 Computational Biology Center, IBM Thomas J. Watson Research Center, Yorktown Heights, NY 10598, USA

## Abstract

Lipid bilayer provides a two-dimensional hydrophobic solvent milieu for membrane proteins in cells. Although the native bilayer is widely recognized as an optimal environment for folding and function of membrane proteins, the underlying physical basis remains elusive. Here, employing the intramembrane protease GlpG of *Escherichia coli* as a model, we elucidate how the bilayer stabilizes a membrane protein and engages the protein’s residue interaction network compared to the nonnative hydrophobic medium, micelles. We find that the bilayer enhances GlpG stability by promoting residue burial in the protein interior compared to micelles. Strikingly, while the cooperative residue interactions cluster into multiple distinct regions in micelles, the whole packed regions of the protein act as a single cooperative unit in the bilayer. Molecular dynamics (MD) simulation indicates that lipids less efficiently solvate GlpG than detergents. Thus, the bilayerinduced enhancement of stability and cooperativity likely stems from the dominant intraprotein interactions outcompeting the weak lipid solvation. Our findings reveal a foundational mechanism in the folding, function, and quality control of membrane proteins. The enhanced cooperativity benefits function facilitating propagation of local structural perturbation across the membrane. However, the same phenomenon can render the proteins’ conformational integrity vulnerable to missense mutations causing conformational diseases^1,2^.

The solvent plays a pivotal role in the folding and function of proteins^3,4^. For water-soluble proteins, the hydrophobic effect (*i.e*., the unfavorable ordering of water molecules around nonpolar solutes) provides a crucial driving force for folding by inducing collapse of nonpolar residues in the protein interior, leading to expulsion of solvating water to the bulk aqueous medium^5^. Involving the collective formation and disruption of water hydrogen (H)-bond networks, the solvent further mediates cooperativity in the folding and allosteric protein–ligand interactions^4,6–9^.

In contrast, membrane proteins fold and function in a lipid bilayer. The folding of helical membrane proteins can be described using the two-stage model^10^: In Stage I, transmembrane (TM) helices are established across the bilayer driven by the hydrophobic effect that induces the burial of nonpolar segments of the polypeptide chain in the bilayer, further benefitted by the favorable formation of backbone H-bonds therein^11,12^. In Stage II, the helices associate into a compact native structure. While the hydrophobic effect is not likely strong within the bilayer due to the lack of water, various molecular forces are known to drive this stage including interhelical van der Waals (vdW) packing and polar interactions^13–16^, the backbone and side-chain entropy^17,18^, and the bilayer forces (*e.g*., the lipid-packing pressure and lipid deformation by the hydrophobic mismatch between the bilayer and protein)^19–21^.

Nonetheless, the precise role of lipid solvation in the folding energy landscape and cooperativity of membrane proteins is poorly understood. The “lipophobic effect” (the lipid-mediated protein association in the membrane) has been proposed as an analogous phenomenon to the hydrophobic effect in water^21–24^, but such effect remains untested for the folding of bona fide membrane proteins. Cooperativity, which links the behavior of distant sites^25^, underlies the function of membrane proteins (*e.g*., receptors and transporters) enabling propagation of physical or chemical stimuli from one side of the protein to the other across the bilayer. It is unknown if lipids play a role in mediating cooperativity for membrane proteins as water does for globular proteins.

Using GlpG, a member of the universally conserved rhomboid protease family, we hypothesize that, if the lipid bilayer simply serves as an inert hydrophobic solvent for membrane proteins, the information pertaining to stability and cooperativity is solely encoded in the amino acid sequence. Consequently, the characteristics of a hydrophobic medium would have no impact on the strengths of individual residue interactions or their overall interaction network. To test this hypothesis, we conducted a comparative analysis on the stability, residue interaction network, and solvation dynamics of GlpG in two distinct hydrophobic media: bicelles (a lipid bilayer edge-stabilized by detergents) composed of 1,2-dimyristoyl-sn-glycero-3-phosphocholine (DMPC) and 3-[(3-cholamidopropyl) dimethylammonio]-1-propanesulfonate (CHAPS), which serves as a native-like bilayer medium, and dodecylmaltoside (DDM) micelles, which represents a nonnative hydrophobic medium (Fig. 1a). These specific amphiphilic assemblies are widely used in structural, folding, and functional studies of membrane proteins^26,27^.

## Bilayer increases GlpG stability.

**Fig. 1│:**
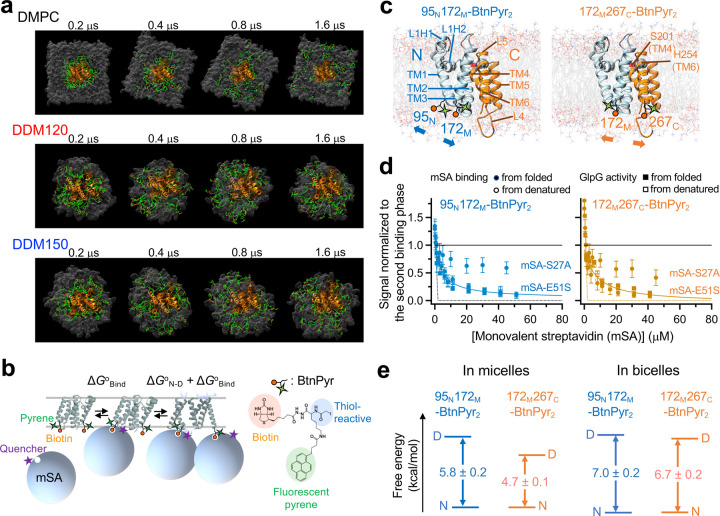
Establishment of the reversible folding of GlpG in bicelles. **(a)** Solvation dynamics of amphiphiles on GlpG in the DMPC bilayer and DDM micelles studied by all-atom MD simulation. The illustration shows the tracking of the 40 lipid or detergent molecules (green) on GlpG (orange) in the first solvation shell at the simulation time, *t* = 0.2 μs. DDM120: the aggregation number (*N*_A_) = 120; DDM150: *N*_A_ = 150. **(b)** Steric trapping scheme. GlpG is labeled with the biotin tags at two specific residues, which are close in space in the native state but distant in the amino acid sequence. A first monovalent streptavidin (mSA, 52 kDa) binds unhindered to either biotin tag (Δ*G*^o^_Bind_). Due to the steric hindrance with the first bound mSA. A second mSA binds only when the tertiary contacts between the biotinylated sites are denatured. The coupling between GlpG denaturation and mSA binding attenuates the apparent second binding (Δ*G*^o^_Bind_ + Δ*G*^o^_N-D_). Δ*G*^o^_N-D_ is determined by fitting the second binding phase to Eqs. 3–5 **(Online Methods)**. **(c)** Crystal structure of GlpG (PDB: 3B45)^37^ annotated with the secondary structural elements, N (cyan)- and C (orange)-subdomains, and the positions of biotin pairs. **(d)** Reversibility of the folding and mSA binding, and their coupling in bicelles. Native (“from folded”) or sterically denatured (“from denatured”) GlpG in micelles before transfer to bicelles at an increasing concentration of mSA-E51S (*K*_d,biotin_ = 52 ± 19 pM) or mSA-S27A (*K*_d,biotin_ = 3.2 ± 0.8 nM). The signals for mSA binding and denaturation were normalized to the amplitude of the second binding phase and the change in activity of GlpG, respectively. Dashed lines: unhindered mSA binding without coupling to denaturation. **(e)** The effect of the bilayer environment on the stabilities of N- and C-subdomains of GlpG. (**d–e**) Errors denote ± SEM (*n* = 3).

Quantification of the thermodynamic stability of a membrane protein (Δ*G*^o^_N-D_, the free energy difference between the native and denatured states) is a daunting task due to the inherent difficulty of achieving the folding reversibility in a lipid bilayer^28^. Here, we overcame the challenge by employing the steric trapping strategy, which capitalizes on the coupling of the spontaneous denaturation of a doubly biotinylated protein to the simultaneous binding of two bulky monovalent streptavidin (mSA) molecules (Fig. 1b)^29–31^. This strategy successfully recapitulates Stage II of membrane protein folding (see below).

Cryo-electron microscopy of bicelles indicates the formation of uniform discoidal bilayers with a diameter of ~90 Å (Extended Data Fig. 1), large enough to accommodate both native and sterically denatured GlpG^32^. To site-specifically biotinylate GlpG, we employed BtnPyr, the thiol-reactive biotin derivative with pyrene (Fig. 1b and Extended Data Fig. 2)^30^. Upon conjugation to GlpG, pyrene fluorescence reports the binding of quencher-labeled mSA^30^. We generated the double-biotin variants, 95_N_172_M_‒BtnPyr_2_ and 172_M_267_C_‒BtnPyr_2_ (N, M, and C: the residue positions of engineered cysteine, the N-terminal, Middle, and C-terminal helices, respectively) to measure the stability at the N- and C-terminal halves of GlpG (*i.e*., N- and C-subdomains), respectively (Fig. 1c). The binding isotherms between GlpG and a weaker biotin-affinity mutant, mSA-E51S (Extended Data Figs. 3–5) displayed a tight, unhindered first binding followed by an optimally attenuated second binding (Figs. 1b and 1d). The inactivation phase of proteolytic activity of GlpG, which was used as a folding indicator, agreed with the second mSA binding phase regardless of whether GlpG was native or sterically denatured prior to incorporation into bicelles (Extended Data Figs. 6–8). Sterically denatured GlpG became highly susceptible to proteolysis by Proteinase K, indicating the increased conformational flexibility and water accessibility relative to native GlpG (Extended Data Fig. 9). This result establishes the folding reversibility and coupling between the second mSA binding and denaturation, thereby validating the steric trapping scheme.

The second mSA binding phases were fitted to yield Δ*G*^o^_N-D,bicelle_^N^ = −7.0 ± 0.2 kcal/mol for N-subdomain and Δ*G*^o^_N-D,bicelle_^C^ = −6.7 ± 0.2 kcal/mol for C-subdomain in bicelles (Fig. 1e). For comparison, the two subdomains exhibit distinct folding properties in micelles: N-subdomain (Δ*G*^o^_N-D,micelle_^N^ = −5.8 ± 0.2 kcal/mol), whose disruption leads to global denaturation, is more stable than C-subdomain (Δ*G*^o^_N-D.micelle_^C^ = −4.7 ± 0.1 kcal/mol), which undergoes subglobal denaturation^30^. Thus, relative to micelles, bicelles stabilized N- and C-subdomains by −1.2 ± 0.3 kcal/mol and −2.0 ± 0.3 kcal/mol, respectively, and induced a near-uniform subdomain stability (|Δ*G*^o^_N-D,bicelle_^N^ – Δ*G*^o^_N-D,bicelle_^C^ | = 0.3 ± 0.3 kcal/mol). The stability that we determined directly under native conditions (Δ*G*^o^_N-D_ = −12*k*_B_*T*) is larger than that obtained from extrapolation to zero force (–6.5*k*_B_*T*) in the molecular tweezer study, likely due to the difference in conformation of the denatured states (Extended Data Fig. 10)^33–35^.

## Bilayer facilitates residue burial.

**Fig. 2│:**
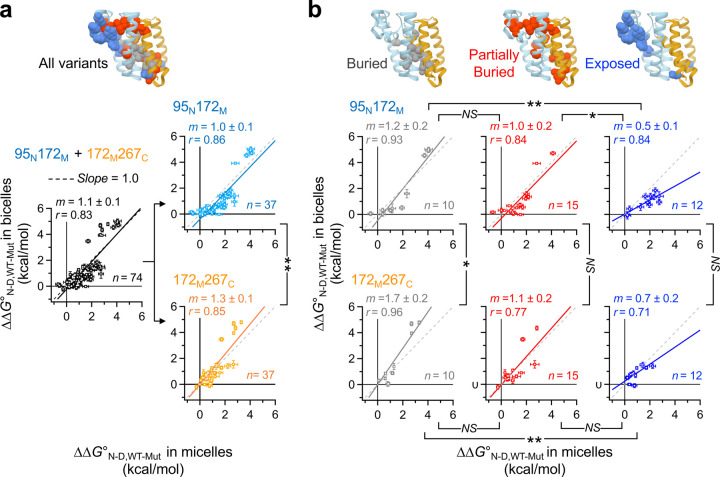
GlpG stability measured at N- and C-subdomains in micelles and bilayers. **(a)** (*Left*) Comparison of the mutation-induced stability changes (ΔΔ*G*^o^_N-D,WT-Mut_’s) in micelles *vs* bicelles. The plot includes all ΔΔ*G*^o^_N-D,WT-Mut_ values measured at N- and C-subdomains. (*Right*) The ΔΔ*G*^o^_N-D,WT-Mut_ values in micelles *vs* bicelles were separately plotted depending on the location of the biotin pair at which the stability was measured. **(b)** Comparison of the ΔΔ*G*^o^_N-D,WT-Mut_ values in micelles *vs* bicelles depending on the location of the biotin pair and the degree of burial of the residues targeted for mutation (*f*_ASA_: the fraction of solvent-accessible residue surface area). **(a–b)** Errors denote ± SD from fitting. The statistical significance of the difference in correlation slope was evaluated using the pairwise Chow’s test (*NS*: *p*>0.05; *: *p*<0.05; **: *p*<0.005)

Next, we investigated whether the features of hydrophobic environment affect the contribution of individual residue interactions to GlpG stability. To this end, 37 residues with various degrees of burial in the protein interior were chosen for large-to-small mutation except for A253V, G261A, and A265V. All mutation-induced stability changes measured at both subdomains in micelles *vs* bicelles (ΔΔ*G*^o^_WT-Mut,micelle_ *vs* ΔΔ*G*^o^_WT-Mut,bicelle_) (Fig. 2a-*left*; Extended Data Tables 1–2; Extended Data Figs. 11–12) displayed linear correlation with the slope close to 1 (*m* = 1.1 ± 0.1). This may indicate that individual residue interactions make a similar contribution to the stability in both environments. However, the mutational impacts displayed differential environmental sensitivity depending on the region where the stability was measured (Fig. 2a-*right*): While the mutations caused destabilization of N-subdomain to a similar extent in micelles and bicelles (*m* = 1.0 ± 0.1), they induced larger destabilization of C-subdomain in bicelles than in micelles (*m* = 1.3 ± 0.1) (*p*<0.005 from Chow’s test).

In-depth analysis of the mutational impacts based on the degree of residue burial of mutated residues provided insights into the origin of the environmental sensitivity. For the mutations of residues completely buried in the protein interior (Fig. 2b-*left*), the fitted slopes exceeded one (*m* = 1.2 ± 0.2 at N-subdomain and *m* = 1.7 ± 0.2 at C-subdomain). Thus, an impact of disrupting internal packing was amplified in bicelles, indicating that the bilayer induced more favorable burial of wild-type residues in the protein interior than micelles. This tendency was more pronounced at C-subdomain than at N-subdomain (*m* = 1.7 *vs* 1.2, *p*<0.05) regardless of the position of mutation. As the degree of residue exposure increased, the slope decreased to *m* = 0.5–0.8 (*p*<0.005).

The differential environmental sensitivity of mutational impacts observed at the two subdomains can be attributed to their distinct packing motifs (Extended Data Fig. 13). In C-subdomain, the stability primarily arises from the backbone–backbone contact between the conserved Gly-zipper motifs (Gly–x_3_–Gly–x_3_–Gly: Gly can be Ala or Ser, and x is any residue) in TM4 and TM6, respectively^30,36–38^. Due to the weakly polar nature of the backbone contact, the bilayer which has a more dehydrated hydrocarbon core than a micelle provides the enhanced stability (Fig. 1e)^39,40^. Thus, disruption of this backbone contact leads to the larger destabilization^31^ (*m* = 1.7). On the other hand, the stability of N-subdomain mainly relies on extensive vdW contacts involving large and small nonpolar residues^30,38^. The impact of disrupting the vdW contacts showed a modest sensitivity to the specific features of the hydrophobic environment (*m* = 1.2).

## Bilayer enhances cooperativity.

Are the lipid effects on GlpG stability localized only to the specific region under investigation (*i.e*., the subdomain or the site of mutation) or do they globally impact the residue interaction network? To address this question, we employed our cooperativity profiling analysis allowing us to investigate whether a given residue engages in local or cooperative interactions with its surrounding^30^. This experimental approach measures the degree of spatial propagation of structural perturbation induced by a point mutation, as quantified by the differential effect of the mutation on the stability of the two subdomains (*i.e*., ΔΔΔ*G* = ΔΔ*G*^o^_WT-Mut_^N^ − ΔΔ*G*^o^_WT-Mut_^C^)^30^. We used four regular cut-off values, ΔΔΔ*G* = −2*RT*, −*RT*, +*RT* and +2*RT* (*R*: gas constant and *T*: absolute temperature) to define the cooperativity profile of each residue^30^.

**Fig. 3│:**
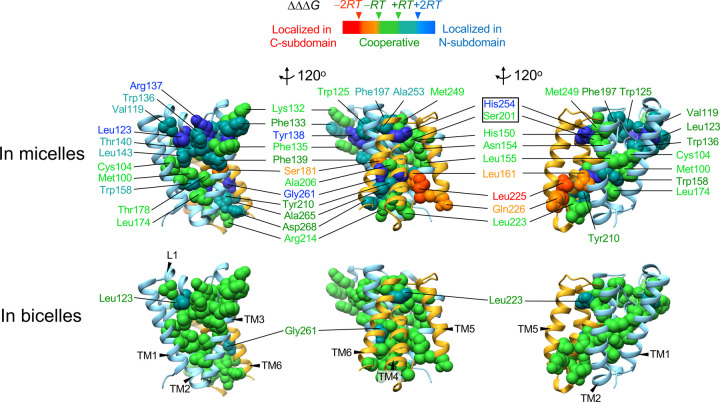
Cooperativity maps of GlpG in micelles and bicelles. The cooperativity profile of each residue is color-coded: “cooperative” (green, |ΔΔΔ*G*|≤*RT* = 0.6 kcal/mol), “moderately localized in N-subdomain” (tin, 2*RT*≥ΔΔΔ*G*>*RT*), “localized in N-subdomain” (blue, ΔΔΔ*G*>2*RT*), “moderately localized in C-subdomain” (orange, −*RT*>ΔΔΔ*G*≥−2*RT*), and “localized in C-subdomain” (red, −2*RT*>ΔΔΔ*G*).

The cooperativity profiles mapped on the GlpG structure unveiled distinct types of residue interactions, classified as “cooperative” (a mutation similarly destabilizes the two subdomains), “localized” (a mutation preferentially destabilizes the subdomain bearing the mutation), and “overpropagated” (a mutation on one subdomain induces the larger destabilization of the other) (Fig. 3). In micelles, cooperative interactions clustered into multiple distinct regions, including the packing core near the bilayer center (Met100, Cys104, Leu174 and Thr178)^30^, the water-conduction channel (Ser201, Met249, His150 and Asn154) connected to the catalytic dyad (Ser201–His254)^41^, and the TM4/TM6 interface where many residues (Ala253, His254, Gly261, Ala265 and Asp268) engaged in overpropagated interactions^30^.

Strikingly, the cooperativity map exhibited a substantially different pattern in bicelles. Most of the localized and overpropagated interactions in micelles turned into cooperative interactions in bicelles. Resultantly, the entire packed regions of GlpG formed a single cooperative unit in bicelles. The narrower cutoff values (ΔΔΔ*G* = −*RT*, −1/2*RT*, +1/2*RT* and +*RT*) yielded the cooperativity profiles resembling those in micelles with the regular cutoff values (Extended Data Fig. 14). Thus, the cooperativity profiles in micelles were partially preserved in bicelles.

This result provides compelling evidence that the bilayer tightly engages the residue interaction network compared to micelles, thereby facilitating propagation of structural perturbation throughout the protein. Thus, the lipid effects on the stability stem from the globally strengthened cooperative network. The proteolytic mechanism of GlpG involves the coordinated motions of multiple structural segments (TM4, TM6, L4 and L5) upon substrate binding^42^. Remarkably, bicelles elicited a five-fold increase in GlpG activity relative to micelles for both membrane-bound (Extended Data Figs 15–16) and water-soluble substrates^43^, which may be attributed to the augmented cooperativity in bicelles.

## Bilayer weakly solvates protein.

Finally, to elucidate the molecular basis underlying the environmental dependence of GlpG stability and cooperativity, we carried out all-atom MD simulation of the GlpG–bilayer and GlpG–micelle complexes, as well as the micelles alone up to 2.3 μs. In the micelle simulation, we chose two aggregation numbers of DDM, *N*_A,DDM_ = 120 (DDM120) and 150 (DDM150) within the experimental range (*N*_A,DDM_ = 90–150)^44^ (Extended Data Fig. 17). Although a comprehensive analysis of protein stability requires the study of both the native and denatured states, modeling of the denatured state ensemble (DSE) in atomic details is yet challenging for membrane proteins. Thus, our simulation focused on the native state to infer the amphiphile effects on the stability.

**Fig. 4│:**
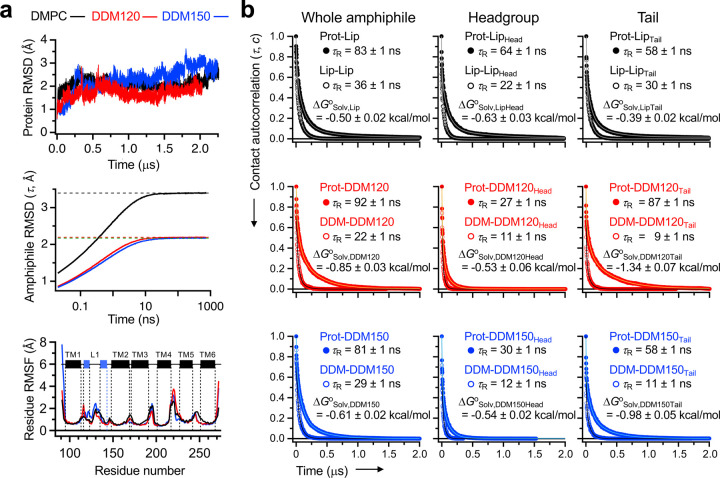
All-atom MD simulation of GlpG WT in the lipid bilayer and micelles. **(a)** The RMSD’s of the backbone heavy atoms (*top*). The RMSD (*τ*) of the lipid or detergent conformation in the bulk amphiphilic phase measured as a function of the time lag *τ* (*middle*). The residue RMSF’s of GlpG WT in each environment (*bottom*). **(b)** The contact autocorrelation on time for measuring the residence time of any heavy atoms in the lipid or detergent molecules on GlpG or on themselves (*left*). The contact autocorrelation was also separately monitored for the heavy atoms in the headgroup (*middle*) or in the tail (*right*) of the amphiphiles. The solid line is the fitted result of each data to a triple-exponential decay function (Extended Data Table 3), implying heterogeneous modes of dissociation. Errors denote ± SD from fitting.

Upon reaching the conformational equilibration of both protein and amphiphiles (Fig. 4a), we analyzed the solvation dynamics of amphiphiles by calculating the contact autocorrelation as a function of time for the protein–amphiphile and amphiphile–amphiphile interactions, respectively, and their residence times (*τ*_R_’s) (Fig. 4b)^45^. The t_R_ was defined as the time at which the amplitude of autocorrelation reached 1/*e* of its initial value. All contact autocorrelation decayed to <1%, indicating that the intermolecular interactions involving amphiphiles were largely equilibrated during simulation. Interestingly, amphiphiles exhibited longer residence times on GlpG (80–90 ns) than on themselves (20–40 ns).

Based on the equilibrated solvation dynamics and the residence times, we were able to quantify the solvation free energy (Δ*G*o_Solv_ = −*RT*•ln[*τ*_R_,_protein-amphiphile_/τ_R_,_amphiphile-amphiphile_]) as a measure of an amphiphile’s affinity for the first solvation shell of GlpG relative to the likewise favorable interaction with another amphiphile in the bulk. Surprisingly, contrary to the expectation that a lipid molecule with double aliphatic tails would form stronger vdW contacts with the protein than a detergent with a single tail, lipids exhibited weaker solvation on GlpG than detergents (Δ*G*^o^_Solv,Lip_ = −0.50 ± 0.02 kcal/mol *vs* Δ*G*^o^_Solv,DDM120_ = −0.85 ± 0.03 kcal/mol and Δ*G*^o^_Solv,DDM150_ = −0.61 ± 0.02 kcal/mol) (Fig. 4b-*left*). This weaker lipid solvation was attributed to the longer *τ*_R_ of lipid–lipid contacts and the comparable or shorter *τ*_R_ of protein–lipid contacts than the respective τ_R_’s of detergents.

Notably, the increase in *N*_A,DDM_ of a micelle from 120 to 150 led to the weakening of solvation possibly due to the increased detergent-mixing entropy in the larger micellar volume. Furthermore, the headgroup and tail regions of an amphiphile differently contributed to the solvation (Fig. 4b-*middle* and *right*). For lipids, the solvation was primarily driven by the headgroup whereas for detergents, by the tail.

MD simulation suggests that the bilayer acts as a poorer solvent than micelles mainly due to the strong lipid–lipid interactions, which facilitate dissociation of lipids from the protein. This leads to the intriguing scenario that the lipid-induced enhancement of stability and cooperativity arises from the dominant intraprotein interactions which outcompete the weak lipid solvation. Despite the small difference in Δ*G*^o^_Solv_ between lipids and detergents (~0.2 kcal/mol), considering the number of amphiphile molecules in the first solvation shell (~40 for lipids and ~70 for detergents), the cumulative difference in Δ*G*^o^_Solv_ piles up to ~30 kcal/mol. This difference can impact the folding energetics of GlpG, highlighted by the dramatic influence of lipid environment on the protein’s stability and cooperativity.

## Conclusion and Outlook.

Our findings demonstrate a profound impact of lipid solvation on the internal organization of a membrane protein. Contrary to the prevalent view as an inert medium, the bilayer actively participates in folding by promoting compaction of polypeptide chains. The bilayer is also known to induce contraction of the DSE of GlpG allowing partial association of the TM helices^32^. Both the studies point to the general lipophobic effect as a key driving force for membrane protein folding bearing an analogy to the hydrophobic effect for water-soluble proteins. The magnitude of the solvation free energy predicted from our simulation (Δ*G*^o^_Solv_ = ~–0.6 kcal/mol-lipid) is comparable to the thermal energy (~0.6 kcal/mol). Thus, protein interactions, either intra- or intermolecular, whose strengths surpass the thermal fluctuation can drive compaction of the polypeptide chains in the bilayer. This suggests that the solvation force as a minimal safety threshold for preventing nonspecific collapse of polypeptide chains may not be strong in the bilayer. Notably, the cellular folding and maturation is known to be inherently inefficient for membrane proteins, rendering the actions of protein quality control mechanisms necessary to alleviate misfolding stresses in all types of subcellular organelles^46^.

The lipid-induced enhancement of cooperativity is a double-edged sword in the function, folding, and quality control of membrane proteins. Many membrane proteins require conformational changes spanning the entire lengths of the proteins to transmit chemical or physical stimuli across the bilayer^47–49^. Our finding indicates that the bilayer serves as an adequate conductive medium of such stimuli, facilitating conformational changes in a cooperative manner. However, the enhanced cooperativity can render the conformational integrity of membrane proteins susceptible to disease-causing mutations (Fig. 2b-*left*). Most of disease-causing mutations on proteins are known to be detrimental to the stability rather than to disrupt active-site residues^2,50,51^. The mapping of disease-causing mutations on the structures of G-protein coupled receptors, ion channels and transporters displays a strong bias of finding such mutations in the TM regions over the extramembraneous regions^1^. In the TM regions, the propensity is even more pronounced for the residues buried in the protein interior than for the residues exposed to the lipid environment^1^. Collectively, our study reveals a foundational physical principle underlying various molecular events that occur in the cell membranes.

## MATERIALS AND METHODS

### Expression and purification of GlpG.

*E. coli* BL21(DE3)RP cells were transformed with pET21a vector encoding the transmembrane (TM) domain (residues 87–276) of GlpG^1^. The cells in Luria-Bertani (LB) broth were grown at 37°C until OD_600nm_ reached 0.9. Protein expression was induced at 0.5 mM IPTG followed by overnight culture at 15°C. Harvested cells were resuspended in 50 mM 2-amino-2-(hydroxymethyl)propane-1,3-diol;dihydrochloride (Tris-HCl) buffer (pH 8.0, 5 mM ethylenediamine-tetraacetic acid (EDTA), 0.5 mM Tris-(2-carboxyethyl)phosphine (TCEP), 0.5 mM phenylmethylsulfonyl fluoride (PMSF)). After the removal of aggregates, the supernatant of cell lysates was centrifuged to isolate the total membrane fraction at 24,000 rpm for 2 h in the 45Ti rotor using ultracentrifuge (Beckman-Coulter). Membrane resuspension in 50 mM Tris-HCl buffer (pH 8.0, 200 mM NaCl, 1 mM TCEP, 0.25 mM PMSF) was solubilized by the addition of 0.7%-w/v *n*-dodecyl-β-D-maltoside (DDM). After the removal of aggregates, GlpG in supernatant was purified using nickel-nitrilotriacetic acid (Ni-NTA, Qiagen) affinity chromatography in 50 mM Tris-HCl buffer (pH 8.0, 200 mM NaCl, 0.1% DDM).

### Biotinylation of GlpG.

50 μM of the double-cysteine variant of GlpG (P95C/G172C or G172C/V267C) was incubated with a 10-molar excess of TCEP for 1 h at 25°C. A 40-molar excess of BtnPyr-IA in dimethyl sulfoxide (DMSO) was added and the reaction proceeded overnight at 25°C. Excess free labels were removed by washing GlpG bound to Ni-NTA resin with 0.1%-w/v DDM and further by dialysis. Labeling efficiency was determined by measuring the absorbance of pyrene (*ε*_346nm_ = 42,000 M^−1^cm^−1^) and protein concentration by 660 nm assay (Bio-Rad). An SDS-PAGE shift assay for isolating single-mSA bound, double-mSA bound, and no-mSA bound GlpG was carried by mixing 10 μl of 5 μM GlpG with 10 μl of the SDS sample buffer (30 min), adding 10 μl of 25 μM mSA-WT (30 min), and running SDS-PAGE on ice without sample boiling.

### Preparation of monovalent streptavidin.

Detailed procedures are described in the previous literatures^1–3^. Streptavidin (active or inactive) encoded in pET21a vector was expressed in *E. coli* BL21(DE3)RP cells in inclusion body. To label mSA with a thiol-reactive dabcyl quencher (dabcyl-maleimide, AnaSpec), Tyr83 near the biotin-binding pocket in the active subunit was mutated to cysteine. Active subunit: wild-type streptavidin or its weaker biotin affinity variants (W79M, S45A, S27A, and E51S) with a C-terminal His_6_ tag; Inactive subunits: the triple mutant (N23A/S27D/S45A) without His_6_ tag^2^.

### Expression and purification of GlpG substrate SN-LYTM2.

The construct containing the second TM segment of *E. coli* lactose permease (LYTM2)^4^ fused to the C-terminus of staphylococcus nuclease (SN) (SN–LYTM2) was encoded in pET30a vector^1^. SN–LYTM2 was expressed and purified using the protocol described previously^1^. In LYTM2, the residue at five residue-upstream of the scissile bond was mutated to cysteine for conjugating the thiol-reactive, environment-sensitive fluorophore, iodoacetyl-7-nitrobenz-2-oxa-1,3-diazol (IA–NBD amide, Setareh Biotech). The initial slope of NBD fluorescence change *vs* time represented proteolytic activity of GlpG. NBD fluorescence were monitored using a SpectraMax M5e plate reader (Molecular Devices) with *λ*_Excitation_ = 485 nm and *λ*_Emission_ = 535 nm.

### Cryo-electron microscopy of bicelles.

3%-w/v 1,2-dimyristoyl-sn-glycero-3-phosphocholine (DMPC)/3-((3-cholamidopropyl)dimethylammonio)-1-propanesulfonate (CHAPS) bicelles ([DMPC]/[CHAPS], *q* = 1.5) was prepared without GlpG. Cryo-EM grids were frozen using a Vitrobot Mark IV (ThermoFisher). Briefly, 3.5 μL of each sample was applied to a glow-discharged Quantifoil Cu 1.2/1.3 holey carbon 200-mesh grid. The grid was blotted for 3.5 s prior to plunge freezing in liquid ethane. Cryo-EM images were recorded on a Talos Arctica (ThermoFisher) operated at 200 kV and equipped with a Falcon 3EC direct electron detector camera. Images were recorded in counting mode using EPU software at a nominal magnification of x92,000 (1.12 Å/pixel), with a defocus of −2.5 μm. Micrographs were collected as single-frame images with a total exposure time of 1.5 s and a total dose of 30 electrons/Å^2^. A total of 6,538 particles from 10 images were auto-picked and extracted into 192 x 192-pixel boxes. The particles were then subjected to 2D classification using cryoSPARC into 50 classes. The diameter of the bicelle in each class average was measured to estimate the size distribution of the bicelles.

### Preparation of native and sterically denatured GlpG in micelles.

20 μM of the double-biotin variants of GlpG (95_N_172_M_–BtnPyr_2_ or 172_M_267_C_–BtnPyr_2_) was incubated with 2.4 molar excess of mSA_DAB_-E51S in 20 mM N-2-hydroxyethylpiperazine-N-2-ethane sulfonic acid (HEPES) buffer (pH 7.5, 40 mM KCl, 0.5 mM DTT, 5 mM DDM) at 25°C. The extent of denaturation was monitored every 24 h using GlpG activity as a folding indicator. For 172_M_267_C_–BtnPyr_2_, maximum denaturation was reached in 24 h. For 95_N_172_M_–BtnPyr_2_, 8 mM SDS was added in the beginning to facilitate the denaturation and incubated for 5 h.

### Fluorescence quenching assay to measure incorporation of GlpG into bicelles.

As a positive control for complete incorporation of GlpG in bicelles, DMPC lipids were mixed with dabcyl–1-palmitoyl-2-oleoyl-sn-glycero-3-phosphoethanolamine (POPE) (Avanti polar lipids) at the molar ratio of 99.5:0.5 in chloroform in a glass tube and dried under stream of nitrogen. After further dried in vacuum for 4 h, the lipid mixture was solubilized in 500 μL of 20 mM HEPES buffer (pH 7.5, 5%-w/v β-octylglucoside (Anatrace)) at the final lipid concentration of 7.5%-w/v. GlpG variant 95_N_172_M_–BtnPyr_2_ or 172_M_267_C_–BtnPyr_2_ in DDM was added to the resuspension and incubated on ice for 30 min. Biobeads (Bio-Rad) were added to remove the detergents in three steps (for each step, 0.2 g/mL of wet Biobeads for 6–12 h at 25°C). Resulting proteoliposomes were extruded using a 0.2 mm pore size membrane. The total phospholipid concentration was measured using an organic phosphate assay. Based on the lipid concentration, CHAPS was added to form bicelles of *q* = 1.5. The protein concentration was measured using a 660 nm assay. As a negative control for no incorporation, water-soluble mSA-E51S/Y83C labeled with *N*-(1-pyrene)maleimide (ThermoFisher) was used. To prepare an experimental sample, native or sterically denatured GlpG (95_N_172_M_–BtnPyr_2_ or 172_M_267_C_–BtnPyr_2_) in DDM was directly injected to the bicelles containing dabcyl-POPE (20 mM HEPES buffer, pH 7.5, 40 mM KCl). In the control and experimental samples, the final concentrations of pyrene labels, DDM, and bicelles were adjusted to 1 μM, 5 mM, and 3%-m/w, respectively. After the incubation of the mixtures at 25°C for 24 h, pyrene fluorescence was measured with *λ*_Excitation_ = 345 nm and *λ*_Emission_ = 390 nm. The degree of quenching, which was related to the degree of GlpG incorporation into bicelles, was determined by the equation, [*F*_Negative control_ – *F*_Experiment_]/[*F*_Negative control_ – *F*_Positive control_] (*F*: fluorescence intensity of pyrene).

### Testing the folding reversibility of GlpG.

Native or sterically denatured GlpG in DDM micelles was directly injected into DMPC/CHAPS bicelles (*q* = 1.5, 3%-w/v) at various concentrations of mSA_Dab_-E51S in 20 mM HEPES buffer (pH 7.5, 40 mM KCl, 0.5 mM DTT) to initiate denaturation and refolding at 25°C, respectively (the final concentration of GlpG = 0.5 μM). To monitor mSA binding, pyrene fluorescence was measured with *λ*_Excitation_ = 345 nm and *λ*_Emission_ = 390 nm on a plate reader every 24 h until an equilibrium was reached (48–72 h). To test the coupling between GlpG denaturation and mSA binding, GlpG activity as a folding indicator was measured at a 20-times molar excess of the substrate incorporated in bicelles.

### Proteinase K digestion of native and sterically denatured GlpG.

Native or sterically denatured GlpG (95_N_172_M_–BtnRG_2_ or 172_M_267_C_–BtnRG_2_) was directly injected into DMPC/CHAPS bicelles (3%-w/v, *q* = 1.5) in 20 mM HEPES buffer (pH 7.5, 40 mM KCl, 5 mM DDM) at the final concentrations of 5 μM and 25 μM for GlpG and mSA-WT, respectively. After incubating at 25°C for 24 h, Proteinase K (the final concentration of 3.4 mg/mL) was added to initiate digestion. The samples were withdrawn at each time point followed by the addition of 10 mM PMSF to quench proteolysis. 10 mM DTT was added and incubated for 1 h to dissociate bound mSA-WT from biotinylated GlpG by cleaving the disulfide bond. SDS-PAGE was run on ice.

### Measuring the intrinsic biotin affinity of mSA variants in bicelles.

The mSA variant with a weaker biotin-binding affinity, mSA_DAB_-W79M (FRET acceptor) was titrated to 100 nM of GlpG singly labeled with BtnPyr (FRET donor) at P95C, G172C, or V267C in 20 mM HEPES buffer (pH 7.5, 3% DMPC/CHAPS bicelles, 40 mM KCl, 0.5 mM DTT). Pyrene fluorescence was measured with *λ*_Excitation_ = 345 nm and *λ*_Emission_ = 390 nm. After 24 h, excess free biotin was added to the final concentration of 2 mM and incubated for another 24 h to dissociate bound mSA. The measured pyrene fluorescence serves as a background. Background-subtracted data were fitted to Eq.1 to obtain *K*_d,biotin_ of mSA_DAB_-W79M in bicelles.^1^

Eq.1 $$F=A1\cdot \frac{\left(P_{T}+[m\text{SA}]+K_{d,\;\text{biotin}\;}\right)-\sqrt{{\left(P_{T}+[m\text{SA}]+K_{d,\;\text{biotin}\;}\right)}^{2}-4P_{T}\cdot [mSA]}}{2P_{T}}+A2$$,

where *F* is the measured fluorescence intensity, *P*_T_ is the total GlpG concentration, [mSA] is the total mSA concentration, *K*_d,biotin_ is the dissociation constant of mSA_DAB_ from biotin, *A*1 is the total net change in fluorescence and *A*2 is the fluorescence level without mSA_DAB_.

The *K*_d,biotin_ of a stronger biotin-binding variants (W79M, S45A, or S27A) was measured by a FRET-based competition assay. 1 μM G172C–BtnPyr was pre-equilibrated with a 2- or 5-times molar excess of the dabcyl-labeled mSA variants for 3 h at 25°C, which represented a quenched state. Next, a weaker biotin-affinity mSA variant without the dabcyl label was titrated against the quenched state. Dequenching of pyrene fluorescence was measured with *λ*_Excitation_ = 345 nm and *λ*_Emission_ = 390 nm. Once equilibrium was reached (24–48 h), 2 mM biotin was added to dissociate bound mSA and further equilibrated for 7–24 h. The fluorescence data served as a background signal. Background-subtracted data were fitted to Eq. 2.

Eq.2 $$F=A1\cdot \frac{-\left[P_{T}+[mSA]+\frac{K_{\text{unlabel}\;}}{K_{\text{label}\;}}\cdot \left(C_{T}-P_{T}\right)\right]+\sqrt{{\left(P_{T}+[mSA]+\frac{K_{\text{unlabel}\;}}{K_{\text{label}}}\cdot \left(C_{T}-P_{T}\right)\right)}^{2}+4P_{T}\cdot [mSA]}\cdot \frac{K_{\text{unlabel}}}{K_{\text{label}}}}{2P_{T}\cdot \frac{K_{\text{unlabel}\;}}{K_{\text{label}}}}+A2$$,

where *K*_unlabel_ is the dissociation constant of mSA without dabcyl from BtnPyr, *K*_label_ is the dissociation constant for mSA_DAB_ from BtnPyr. Fitted values include *A*1, *A*2, and *K*_unlabel_ or *K*_label_. For mSA_DAB_-E51S, 1.5 μM G172C–BtnPyr was first titrated with various concentrations of mSA-S27A without a label, and pyrene fluorescence was measured. This signal served as a background. Then, 2 μM of mSA_DAB_-E51S was added, and quenching of pyrene fluorescence was measured. After reaching an equilibrium (48–72 h), background-subtracted data were fitted to Eq. 2.

### Construction of binding isotherms to determine Δ*G*^o^_N-D_ of GlpG.

1 μM of GlpG (95_N_172_M_–BtnPyr_2_ or 172_M_267_C_–BtnPyr_2_) was titrated with various concentrations of mSA_DAB_ in 20 mM HEPES buffer (pH 7.5, 3%-w/v bicelles or 5 mM DDM micelles, 40 mM KCl, 1 mM DTT). Depending on the stability of GlpG mutant, a series of mSA variants with a weaker biotin affinity (mSA_DAB_-W79M, S45A, S27A, and E51S) were screened until an optimal second binding phase was obtained. The titrated samples were transferred to a 96 well plate, sealed with a polyolefin tape, and incubated at 25°C. After the equilibrium was reached, the binding was measured by quenching of pyrene fluorescence with *λ*_Excitation_ = 345 nm and *λ*_Emission_ = 390 nm on a plate reader. Data were averaged from three fluorescence readings. The second attenuated binding phase was fitted to Eq.4.

### Fitting of the second binding phase to obtain D*G*^o^_N-D_ of GlpG.

The attenuated second binding of mSA was fitted to the equation derived from the following scheme^1^:

Eq.3 $$N\cdot mSA\overset{K_{D}}{⇄}D\cdot mSA\;\text{where}\;K_{D}=\frac{\left[D\cdot mSA\right]}{\left[N\cdot mSA\right]}\;D\cdot mSA+mSA\underset{K_{d,\;\text{biotin}\;}}{⇄}D\cdot 2mSA\;\text{where}\;K_{d,\;\text{biotin}\;}=\frac{\left[D\cdot mSA][mSA\right]}{\left[D\cdot 2mSA\right]}$$

The fitting equation was:

Eq.4 $$F=\frac{1}{\left[1+\left(K_{d,\;\text{biotin}\;}+\frac{K_{d,\;\text{biotin}\;}}{K_{D}}\right)\cdot \frac{1}{\left[mSA\right]}\right]}\cdot \left(F_{\infty }-F_{o}\right)+F_{o}$$

Eq.5 $$\Delta G_{N-D}^{o}=-RT\cdot \text{ln}\left(\frac{1}{K_{D}}\right)$$,

where *F* is the measured fluorescence intensity and *F*_o_ and *F*_∞_ are the fluorescence intensities from BtnPyr conjugated to GlpG at [mSA] = 0 and at [mSA] = ∞, respectively. [mSA] is the total mSA concentration, *K*_d,biotin_ is the unhindered biotin affinity of mSA, and *K*_D_ is the equilibrium constant for denaturation of GlpG. After obtaining the fitted *K*_D_, D*G*^o^_N-D_ was calculated.

### Cooperativity profiling^1^.

Specific residue interaction is perturbed by a single point mutation in the background of the double-biotin variants 95_N_172_M_–BtnPyr_2_ (Δ*G*^o^_N-D_^N^) or 172_M_267_C_–BtnPyr_2_ (Δ*G*^o^_N-D_^C^), which is set as ‘WT’. Then, the stability change induced by the same point mutation was measured by steric trapping for each WT background (ΔΔ*G*^o^_N-D,WT-Mut_^N^ = Δ*G*^o^_N-D,WT_^N^− Δ*G*^o^_N-D,Mut_^N^ or ΔΔ*G*^o^_N-D,WT-Mut_^C^ = Δ*G*^o^_N-D,WT_^C^− Δ*G*^o^_N-D,Mut_^C^). Then, the differential effect of the mutation on the stability of the two subdomains is quantified as follows:

Eq.6 $$\Delta \Delta \Delta G=\left[\Delta G_{N-D,WT}^{o}{}^{N}-\Delta G_{N-D,Mut}^{o}{}^{N}\right]-\left[\Delta G_{N-D,WT}^{o}{}^{C}-\Delta G_{N-D,Mut}^{o}{}^{C}\right]\;=\Delta \Delta G_{N-D,WT-Mut}^{o}{}^{N}-\Delta \Delta G_{N-D,WT-Mut}^{o}{}^{C}$$

We apply four cut-off values, ΔΔΔ*G* = −2*RT*, −*RT*, *RT* and 2*RT* (*i.e*., five sets of the cooperativity profile; *R*: gas constant and *T*: absolute temperature) to resolve the degree of cooperativity of each residue interaction. For a given ΔΔΔ*G* value, we assign the cooperativity profile as follows. +2*RT<*ΔΔΔ*G*: highly localized in N-subdomain; +*RT*<ΔΔΔ*G*≤+2*RT*: moderately localized in N-subdomain; −*RT*≤ΔΔΔ*G*≤+*RT*: cooperative; −2*RT*≤ΔΔΔ*G*≤–*RT*: moderately localized in C-subdomain; ΔΔΔ*G*<–2*RT*: highly localized in C-subdomain.

### Molecular dynamics simulation.

MD simulation setups were based on the crystal structure of *E. coli* GlpG (PDB code: 2IC8). The bicelle was approximated to a lipid bilayer composed of 315 DMPC molecules, which was constructed using the CHARMM-GUI membrane builder^5^. Two micellar systems were set up with 120 (DDM120) and 150 DDM (DDM150) molecules per micelle, modeled by symmetrically enclosing the TM domain of GlpG with DDM molecules. Each of the GlpG–bilayer and GlpG–micelle composite systems were immersed in the TIP3P water solvent^6^ followed by charge neutralization and ionization with 150 mM NaCl. Each system was composed of >90,000 atoms in a 115×115×89 Å^3^ box. Independently, we prepared the DDM120 and DDM150 micelles without GlpG as controls.

All inter- and intramolecular interactions were enumerated under the CHARMM36 force field^7^. The nonbonding van der Waals and short-range electrostatic interactions were treated with a typical cutoff distance of 12 Å, while the long-range electrostatic contributions were evaluated with the particle-mesh Ewald method^8^. All simulations were carried out using GROMACS software^9^ parallelized in the GPU-accelerated IBM Power8 machine. Each system was first subject to 10,000 steps of conjugate gradient energy minimization to remove any unfavorable atomic crash with lipids and GlpG, which were restrained to preserve their conformation and relative positions. The systems were pre-equilibrated along 6 scheduled steps as gradually removing the external restraints until no constraints. The simulations were proceeded with a 2-fs timestep in the semi-isotropic isobaric and isothermal (NPT) ensemble of 1 atm and 310 K, where the pressure and temperature were controlled by Parrinello-Rahman barostat^10^ and Nosé-Hoover thermostat^11,12^, respectively. The pressure was decoupled between the *xy*-plane and the z-axis, so the membrane normal fluctuated independently from the isotropic lateral motions (*xy*-plane). >2 μs-long simulation trajectories were generated for each system.

### Assessment of the equilibration of protein and amphiphiles.

The equilibration of systems was evaluated for: (1) the conformation of protein, (2) the conformation of amphiphiles, and (3) the solvation dynamics of interfacial amphiphiles on protein. The equilibration of GlpG conformation was examined by calculating the RMSD’s of all heavy atoms referenced to the crystal structure^13^. Regarding the equilibration of amphiphile conformation, we assessed the time-autocorrelated RMSD (τ) as a function of the time lag τ by averaging over all lipid or detergent molecules in the bulk (*i.e*., not in contact with the protein) as follows:

Eq.7 $$\text{RMSD}(\tau )=\frac{1}{N_{L}}\overset{N_{L}}{\underset{i=1}{\sum }}<RMSD_{i}{(t,t+\tau )>}_{t}$$,

where *N*_*L*_ is the number of the lipid or detergent molecules, and <*RMSD*_*i*_ (*t*, *t* + *τ*)>_*t*_ is the heavy-atom RMSD between the *i*-th amphiphile’s conformations at the time *t* and *t* + *τ*, averaged over all available time *t’*s. The bulk lipid molecules were selected from the ones not in contact with the protein over the analysis period while the bulk detergent molecules from the control micelles without GlpG. Prior to the RMSD calculation, the amphiphiles under comparison at each *t* and *t* + *τ* were structurally aligned with each other by transrotating the heavy-atom conformations.

For the solvation dynamics of interfacial amphiphiles on protein, we analyzed how fast the lipid (or detergent) would dissociate from the protein by measuring the residence time (*τ*_R_) from the autocorrelation function on time for the amphiphile heavy atom in contact (distance cutoff of 5 Å) with GlpG as follows:^13–15^

Eq.8 $$c(\tau )=\frac{1}{Nc}\overset{Nc}{\underset{i=1}{\sum }}<c_{i}{(t,t+\tau )>}_{t}$$,

where *N*_*c*_ is the number of contact events, and a single contact event is defined as a consecutive contact of an amphiphile with no non-contacting time gap longer than the amphiphile relaxation time measured above (*i.e*., 20 ns). The autocorrelation function at the time τ of the *i*-th contact event, <*c*_*i*_ (*t*, *t* + *τ*)>_*t*_ is defined by <*q*_*i*_(*t*)•*q*_*i*_(*t* + *τ*)/*q*_*i*_^2^(*t*)>_*t*_ the normalized product of heavy atom contact numbers of an amphiphile in the *i*-th contact event, *q*_*i*_(*t*) and *q*_*i*_(*t + τ*) at two time moments (*t* and *t+ τ*), averaged over the time *t*.

### Residence time of amphiphiles.

The *τ*_R_ was defined as the time when the amplitude of contact autocorrelation reached 1/*e* of the initial value without any assumption on the dissociation mechanisms based on Eq.8. Dissociation dynamics of an amphiphile from GlpG was assessed as a whole or parts (*i.e*., head group and tail). The tail of DMPC was defined as the atoms in two aliphatic chains (C22–C214 and C32–C314) while that of DDM included all carbons in the dodecyl chain (C1–C12), thus leaving the rest as the head group. As a control, we assessed self-dissociation of an amphiphile from another amphiphile, where the pairs were selected from the molecules in contact with each other in the bulk. DMPC molecules were selected from ones of no explicit protein contact in the bilayer, and DDM molecules from the micelles without GlpG. In each system, the residence time for self-dissociation was served as a reference for evaluating the relative preference toward protein.

### Solvation free energy (Δ*G*^o^_Solv_) of amphiphiles on GlpG.

Δ*G*^o^_Solv_ was derived from the following equations:

Eq.9 $$P+L\cdot L\underset{k_{off,P\cdot L}}{\overset{k_{off,L\cdot L}}{⇄}}P\cdot L+L$$

Eq.10 $$\Delta G_{\text{Solv}}^{o}=-RT\text{ln}K_{\text{Solv}}=-RT\text{ln}\left(\frac{k_{off\;,L\cdot L}}{k_{off\;,P\cdot L}}\right)=-RT\left(\frac{\tau_{R,P\cdot L}}{\tau_{R,L\cdot L}}\right)$$

*P* and *L*•*L* denote the protein and the amphiphile–amphiphile complex outside of the first solvation shell (*i.e*., in the bulk), respectively. *P*•*L* denotes the protein–amphiphile complex. *k*_off,P•L_ and *k*_off,L•L_ are the dissociation rates of an amphiphile from the protein–amphiphile and amphiphile–amphiphile complexes (*i.e*., the inverse of *τ*_R_), respectively. A negative Δ*G*^o^_Solv_ indicates that an amphiphile favorably makes a contact with protein (τ_R,P•L_) overcoming the likewise favorable contacts between the amphiphiles in the bulk (τ_R,L•L_).

## Supplementary Material

Supplement 1
