# Supplementary material for: Lipid Bilayer Strengthens the Cooperative Network of a Membrane-Integral Enzyme
Source: bioRxiv. 2023 May 31:2023.05.30.542905. Preprint. [Version 1] doi: 10.1101/2023.05.30.542905 (PMC10312574; doi:10.1101/2023.05.30.542905)
Supplement: Supplement 1 [file media-1.pdf]

Supporting Information for:

Lipid Bilayer Strengthens the Cooperative Network of a Membrane-Integral Enzyme

Shaima Muhammednazaar[1], Jiaqi Yao[1], Ruiqiong Guo[1], May S. Rhee[1], Kelly H. Kim[2], Seung-gu Kang[3], and Heedeok Hong[1,2]

[1]Department of Chemistry and [2]Department of Biochemistry & Molecular Biology, Michigan State University, East Lansing, MI 48824, USA

[3]Computational Biology Center, IBM Thomas J. Watson Research Center, Yorktown Heights, NY 10598, USA

To whom correspondence may be addressed: honghd@msu.edu and sgkang@us.ibm.com

Extended Data Table 1: The mutation-induced change in thermodynamic stability ($\Delta\Delta G^{o}_{WT\text{-}Mut}$) and the activity relative to wild type in DMPC/CHAPS bicelles. The fraction of residue buried area (f_{ASA}) and cooperativity profile of each residue. "N" and "C" in the "Location" column denote N- and C-subdomains, respectively. In the "Cooperativity profile" column, "Moderate/N", "Moderate/C", and "Moderate/Over" denote moderately localized in N and C subdomains, and moderately overpropagated, respectively.

Secondary structure	Mutation	**Location**	f_{ASA}	N-subdomain (95_N172_M)		C-subdomain (172_M267_C)		$\Delta\Delta\Delta G^{o}$	Cooperativity profile
				$\Delta\Delta G^{o}_{WT\text{-}Mut}$	Rel. Activity	$\Delta\Delta G^{o}_{WT\text{-}Mut}$	Rel. Activity		
TM1	M100A	N	0.14	1.4 ± 0.2	0.86 ± 0.09	1.4 ± 0.2	0.74 ± 0.08	0.0 ± 0.2	Cooperative
	C104A	N	0	0.5 ± 0.3	0.84 ± 0.09	0.1 ± 0.4	0.58 ± 0.06	0.4 ± 0.4	Cooperative
L1	V119A	N	0.05	1.6 ± 0.2	0.59 ± 0.07	1.1 ± 0.1	0.70 ± 0.07	0.5 ± 0.2	Cooperative
	L123A	N	0.23	1.4 ± 0.1	0.57 ± 0.06	0.7 ± 0.2	0.65 ± 0.07	0.7 ± 0.2	Moderate/N
	W125A	N	0.09	3.9 ± 0.1	0.17 ± 0.02	3.5 ± 0.1	0.12 ± 0.01	0.5 ± 0.1	Cooperative
	K132A	N	0.56	-0.1 ± 0.1	0.79 ± 0.09	0.0 ± 0.1	0.72 ± 0.08	-0.1 ± 0.2	Cooperative
	F133A	N	0.80	0.6 ± 0.1	0.83 ± 0.10	0.8 ± 0.2	0.82 ± 0.09	-0.2 ± 0.2	Cooperative
	F135A	N	0.73	0.4 ± 0.1	1.02 ± 0.12	0.5 ± 0.1	0.75 ± 0.09	-0.1 ± 0.2	Cooperative
	W136A	N	0.46	1.0 ± 0.3	0.22 ± 0.02	1.3 ± 0.1	0.30 ± 0.03	-0.3 ± 0.3	Cooperative
	R137A	N	0.04	4.7 ± 0.1	0.01 ± 0.01	4.3 ± 0.2	0.01 ± 0.01	0.4 ± 0.2	Cooperative
	Y138F	N	0.25	0.9 ± 0.1	0.75 ± 0.08	0.8 ± 0.2	0.56 ± 0.07	0.1 ± 0.2	Cooperative
	F139A	N	0.38	0.7 ± 0.2	0.63 ± 0.07	1.3 ± 0.1	0.45 ± 0.05	0.5 ± 0.2	Cooperative
	T140A	N	0.20	1.4 ± 0.1	0.59 ± 0.07	0.9 ± 0.2	0.62 ± 0.07	0.5 ± 0.2	Cooperative
	L143A	N	0.25	1.3 ± 0.1	0.78 ± 0.09	1.5 ± 0.2	0.68 ± 0.08	-0.2 ± 0.2	Cooperative
TM2	H150A	N	0.01	0.3 ± 0.2	0.10 ± 0.01	0.5 ± 0.2	0.03 ± 0.00	-0.2 ± 0.3	Cooperative
	N154A	N/Interface	0	0.5 ± 0.1	0.12 ± 0.01	0.6 ± 0.1	0.04 ± 0.09	-0.1 ± 0.1	Cooperative
	L155A	N	0.15	1.4 ± 0.1	0.64 ± 0.07	0.9 ± 0.1	0.79 ± 0.08	0.5 ± 0.2	Cooperative
	W158F	N/Interface	0	0.3 ± 0.1	0.84 ± 0.10	0.3 ± 0.1	0.82 ± 0.09	0.0 ± 0.2	Cooperative
	L161A	N/Interface	0	1.2 ± 0.1	0.36 ± 0.04	1.5 ± 0.3	0.26 ± 0.03	-0.3 ± 0.3	Cooperative
TM3	L174A	N/Interface	0	4.5 ± 0.2	0.49 ± 0.05	4.8 ± 0.1	0.17 ± 0.02	-0.3 ± 0.2	Cooperative
	T178A	N	0.11	0.7 ± 0.1	0.77 ± 0.09	0.9 ± 0.2	0.66 ± 0.07	-0.2 ± 0.2	Cooperative
	S181A	N/Interface	0	0.1 ± 0.2	0.92 ± 0.11	0.5 ± 0.1	0.85 ± 0.09	-0.4 ± 0.2	Cooperative
	F197A	N	0.01	0.6 ± 0.2	0.02 ± 0.00	0.5 ± 0.2	0.00 ± 0.00	0.1 ± 0.2	Cooperative
TM4	S201T	C/Interface	0	0.0 ± 0.2	0.05 ± 0.01	0.0 ± 0.2	0.01 ± 0.00	0.0 ± 0.3	Cooperative
	A206G	C	0	0.3 ± 0.2	1.07 ± 0.12	0.9 ± 0.1	0.92 ± 0.10	-0.6 ± 0.2	Cooperative
	L207A	C/Interface	0	5.0 ± 0.2	0.12 ± 0.01	4.7 ± 0.1	0.16 ± 0.02	0.3 ± 0.2	Cooperative
	Y210F	C	0	0.5 ± 0.2	0.86 ± 0.10	0.9 ± 0.1	0.61 ± 0.07	-0.4 ± 0.2	Cooperative
	R214A	C/Interface	0.10	0.2 ± 0.2	0.70 ± 0.08	0.7 ± 0.2	0.52 ± 0.05	-0.5 ± 0.3	Cooperative
TM5	I223A	C/Interface	0.00	0.1 ± 0.2	0.56 ± 0.06	0.6 ± 0.2	0.30 ± 0.03	-0.6 ± 0.2	Moderate/C
	L225A	C/Interface	0.03	0.2 ± 0.1	0.42 ± 0.05	0.4 ± 0.1	0.26 ± 0.03	-0.3 ± 0.1	Cooperative
	Q226A	C	0.57	0.0 ± 0.1	0.95 ± 0.11	-0.1 ± 0.2	0.67 ± 0.07	0.1 ± 0.2	Cooperative
TM6	M249A	C/Interface	0.01	0.3 ± 0.1	0.97 ± 0.11	0.4 ± 0.2	0.79 ± 0.09	-0.1 ± 0.2	Cooperative
	A253V	C/Interface	0	0.0 ± 0.1	0.03 ± 0.01	0.3 ± 0.2	0.03 ± 0.00	-0.3 ± 0.2	Cooperative
	H254A	C	0	0.7 ± 0.1	0.05 ± 0.01	-0.2 ± 0.2	0.01 ± 0.00	0.9 ± 0.2	Moderate/Over
	G261A	C	0	4.9 ± 0.1	0.05 ± 0.01	4.0 ± 0.1	-0.02 ± 0.01	1.0 ± 0.2	Moderate/Over
	A265V	C	0	1.6 ± 0.3	0.37 ± 0.04	1.4 ± 0.2	0.29 ± 0.03	0.2 ± 0.4	Cooperative
	D268A	C/Interface	0.15	1.8 ± 0.2	0.54 ± 0.06	1.5 ± 0.1	0.48 ± 0.05	0.3 ± 0.2	Cooperative

Extended Data Table 2: Extended Data Table 1: The mutation-induced change in thermodynamic stability ($\Delta\Delta G^{o}_{WT\text{-}Mut}$) and the activity relative to wild type in DDM micelles. The fraction of residue buried area (f_{ASA}) and cooperativity profile of each residue. "N" and "C" in the "Location" column denote N- and C-subdomains, respectively. In the "Cooperativity profile" column, "Local/N", "Local/C", "Moderate/N", "Moderate/C", and "Moderate/Over" denote localized in N and C subdomains, moderately localized in N and C subdomains, and moderately overpropagated, respectively.

Secondary structure	Mutation	**Location**	f_{ASA}	N-subdomain (95_N172_M)		C-subdomain (172_M267_C)		$\Delta\Delta\Delta G^{o}$	Cooperativity profile
				$\Delta\Delta G^{o}_{WT\text{-}Mut}$	Rel. Activity	$\Delta\Delta G^{o}_{WT\text{-}Mut}$	Rel. Activity		
TM1	M100A	N	0.14	3.0 ± 0.3	0.55 ± 0.06	2.2 ± 0.3	0.64 ± 0.05	0.5 ± 0.4	Cooperative
	C104A	N	0	1.2 ± 0.3	0.69 ± 0.04	0.9 ± 0.1	0.70 ± 0.05	0.3 ± 0.3	Cooperative
L1	V119A	N	0.05	2.0 ± 0.2	0.57 ± 0.06	0.9 ± 0.1	0.76 ± 0.05	1.1 ± 0.2	Moderate/N
	L123A	N	0.23	2.1 ± 0.2	0.55 ± 0.06	0.9 ± 0.1	0.68 ± 0.05	1.2 ± 0.2	Local/N
	W125A	N	0.09	2.8 ± 0.3	0.04 ± 0.10	1.7 ± 0.2	0.00 ± 0.01	1.1 ± 0.3	Moderate/N
	K132A	N	0.56	0.2 ± 0.3	0.71 ± 0.06	0.4 ± 0.1	0.52 ± 0.05	-0.2 ± 0.3	Cooperative
	F133A	N	0.80	1.3 ± 0.3	0.84 ± 0.06	0.5 ± 0.2	0.87 ± 0.05	0.8 ± 0.3	Cooperative
	F135A	N	0.73	0.4 ± 0.2	0.93 ± 0.06	0.2 ± 0.1	0.69 ± 0.05	0.2 ± 0.3	Cooperative
	W136A	N	0.46	2.7 ± 0.2	0.00 ± 0.02	1.7 ± 0.1	0.00 ± 0.03	1.0 ± 0.2	Moderate/N
	R137A	N	0.04	4.1 ± 0.2	0.01 ± 0.01	2.8 ± 0.1	0.01 ± 0.01	1.3 ± 0.2	Local/N
	Y138F	N	0.25	1.8 ± 0.2	0.95 ± 0.06	0.6 ± 0.1	0.93 ± 0.05	1.2 ± 0.2	Local/N
	F139A	N	0.38	2.0 ± 0.2	0.47 ± 0.06	1.0 ± 0.1	0.47 ± 0.05	1.0 ± 0.2	Moderate/N
	T140A	N	0.20	1.6 ± 0.2	0.85 ± 0.06	0.7 ± 0.1	0.60. ± 0.03	0.9 ± 0.2	Moderate/N
	L143A	N	0.25	2.3 ± 0.2	0.76 ± 0.06	1.4 ± 0.1	0.65 ± 0.05	0.9 ± 0.2	Moderate/N
TM2	H150A	N	0.01	0.0 ± 0.3	0.05 ± 0.08	0.3 ± 0.2	0.02 ± 0.13	-0.3 ± 0.3	Cooperative
	N154A	N/Interface	0	1.2 ± 0.2	0.01 ± 0.04	1.2 ± 0.3	0.01 ± 0.02	0.0 ± 0.4	Cooperative
	L155A	N	0.15	2.2 ± 0.2	0.75 ± 0.05	1.6 ± 0.2	0.60 ± 0.03	0.6 ± 0.3	Cooperative
	W158F	N/Interface	0	1.0 ± 0.2	0.92 ± 0.06	0.1 ± 0.1	0.85 ± 0.05	0.9 ± 0.2	Moderate/N
	L161A	N/Interface	0	2.0 ± 0.3	0.16 ± 0.06	2.7 ± 0.3	0.10 ± 0.06	-0.7 ± 0.4	Moderate/C
TM3	L174A	N/Interface	0	3.7 ± 0.2	0.35 ± 0.06	3.3 ± 0.1	0.07 ± 0.07	0.4 ± 0.2	Cooperative
	T178A	N	0.11	0.6 ± 0.2	0.77 ± 0.06	0.3 ± 0.1	0.66 ± 0.07	0.3 ± 0.2	Cooperative
	S181A	N/Interface	0	-0.6 ± 0.2	1.03 ± 0.06	0.6 ± 0.1	1.00 ± 0.05	-1.2 ± 0.2	Moderate/Over
	F197A	N	0.01	1.7 ± 0.2	0.01 ± 0.03	0.6 ± 0.1	0.00 ± 0.07	1.1 ± 0.2	Moderate/N
TM4	S201T	C/Interface	0	0.4 ± 0.2	0.02 ± 0.01	0.8 ± 0.2	0.00 ± 0.03	-0.4 ± 0.3	Cooperative
	A206G	C	0	0.4 ± 0.2	0.09 ± 0.09	0.6 ± 0.1	0.09 ± 0.06	-0.2 ± 0.2	Cooperative
	L207A	C/Interface	0	4.1 ± 0.3	0.12 ± 0.01	2.7 ± 0.1	0.16 ± 0.02	1.4 ± 0.3	Local/N
	Y210F	C	0	1.9 ± 0.2	0.50 ± 0.07	1.2 ± 0.1	0.66 ± 0.05	0.8 ± 0.2	Moderate/N
	R214A	C/Interface	0.10	0.9 ± 0.2	0.41 ± 0.06	0.6 ± 0.1	0.43 ± 0.05	0.3 ± 0.3	Cooperative
TM5	I223A	C/Interface	0.00	1.0 ± 0.3	0.24 ± 0.06	0.5 ± 0.1	0.23 ± 0.11	0.5 ± 0.3	Cooperative
	L225A	C/Interface	0.03	-0.7 ± 0.2	0.27 ± 0.07	1.0 ± 0.1	0.10 ± 0.06	-1.6 ± 0.2	Local/C
	Q226A	C	0.57	0.2 ± 0.2	0.82 ± 0.06	0.8 ± 0.2	0.51 ± 0.05	-0.6 ± 0.3	Moderate/C
TM6	M249A	C/Interface	0.01	0.3 ± 0.2	0.59 ± 0.06	0.5 ± 0.2	0.85 ± 0.05	-0.2 ± 0.3	Cooperative
	A253V	C/Interface	0	1.5 ± 0.2	0.06 ± 0.01	0.9 ± 0.1	0.00 ± 0.06	0.6 ± 0.3	Moderate/Over
	H254A	C	0	1.5 ± 0.2	0.05 ± 0.01	-0.3 ± 0.1	0.01 ± 0.05	1.8 ± 0.3	Over
	G261A	C	0	4.0 ± 0.2	0.05 ± 0.01	2.7 ± 0.1	-0.01 ± 0.06	1.3 ± 0.2	Over
	A265V	C	0	2.3 ± 0.2	0.30 ± 0.06	1.3 ± 0.1	0.13 ± 0.05	1.0 ± 0.2	Moderate/Over
	D268A	C/Interface	0.15	2.4 ± 0.2	0.44 ± 0.07	1.3 ± 0.1	0.28 ± 0.05	1.1 ± 0.2	Moderate/Over

Extended Data Table 3. Fitted parameters of the time-dependent contact autocorrelation data to a triple exponential decay function for the whole (**a**), headgroup (**b**), and tail (**c**) regions of the lipid (Lip) or detergent (Det) molecules on GlpG (Prot) and on themselves. A: % amplitude; τ_R: residence time; $\langle\tau_R\rangle$: the amplitude-weighted average residence time; Adj-R^2: the adjusted R-square; ΔG^o_{SolvEx}: the solvation free energy of an amphiphile molecule on the protein. $\tau_{R,1/e}$ denotes the resident time at which the contact autocorrelation decays to 1/e of the initial value.

a

Whole	A_1 (%)	$\tau_{R,1}$ (ns)	A_2 (%)	$\tau_{R,2}$ (ns)	A_3 (%)	$\tau_{R,3}$ (ns)	A_∞ (%)	$\langle\tau_R\rangle$ (ns)	Adj-R^2	$\tau_{R,1/e}$ (ns)
Lip–Lip	14.2 ± 0.4	1.3 ± 0.1	39.8 ± 0.5	21 ± 1	46.0 ± 0.5	82 ± 1	0.0 ± 0.0	46 ± 1	0.999	36 ± 1
Prot–Lip	29.4 ± 0.6	19 ± 1	54.3 ± 0.5	112 ± 2	11.1 ± 0.6	527 ± 30	1.3 ± 0.1	132 ± 7	0.999	83 ± 1
$\Delta G^o_{Solv,Lip}$ = −0.62 ± 0.03 kcal/mol from $\langle\tau_R\rangle$				$\Delta G^o_{Solv,Lip}$ = −0.50 ± 0.02 kcal/mol from $\tau_{R,1/e}$						
Det–Det120	30.5 ± 0.9	4.1 ± 0.2	50.3 ± 1.3	28 ± 1	18.1 ± 1.8	85 ± 4	0.0 ± 0.0	31 ± 2	0.998	22 ± 1
Prot–Det120	23.8 ± 0.4	4.1 ± 0.1	35.8 ± 0.3	58 ± 1	39.7 ± 0.3	302 ± 2	0.4 ± 0.0	143 ± 2	0.999	92 ± 1
$\Delta G^o_{Solv,Det120}$ = −0.90 ± 0.05 kcal/mol from $\langle\tau_R\rangle$				$\Delta G^o_{Solv,Det120}$ = −0.85 ± 0.03 kcal/mol from $\tau_{R,1/e}$						
Det–Det150	16.6 ± 1.5	3.2 ± 0.4	39.4 ± 1.2	18 ± 1	43.7 ± 1.5	66 ± 1	0.0 ± 0.0	37 ± 2	0.998	29 ± 1
Prot–Det150	25.8 ± 0.3	6.2 ± 0.1	48.6 ± 0.4	83 ± 1	24.1 ± 0.5	307 ± 4	0.1 ± 0.0	118 ± 3	0.999	81 ± 1
$\Delta G^o_{Solv,Det150}$ = −0.69 ± 0.03 kcal/mol from $\langle\tau_R\rangle$				$\Delta G^o_{Solv,Det150}$ = −0.61 ± 0.02 kcal/mol from $\tau_{R,1/e}$						

b

Headgroup	A_1 (%)	$\tau_{R,1}$ (ns)	A_2 (%)	$\tau_{R,2}$ (ns)	A_3 (%)	$\tau_{R,3}$ (ns)	A_∞ (%)	$\langle\tau_R\rangle$ (ns)	Adj-R^2	$\tau_{R,1/e}$ (ns)
Lip–Lip	37.0 ± 0.5	2.2 ± 0.1	31.9 ± 2.4	27 ± 1	31.1 ± 2.6	64 ± 2	0.0 ± 0.0	29 ± 3	0.999	22 ± 1
Prot–Lip	33.1 ± 0.4	8.9 ± 0.2	51.8 ± 0.3	104 ± 1	9.8 ± 0.3	725 ± 39	0.5 ± 0.1	135 ± 6	0.998	64 ± 1
$\Delta G^o_{SolvEx,LipHead}$ = −0.91 ± 0.06 kcal/mol from $\langle\tau_R\rangle$				$\Delta G^o_{SolvEx,LipHead}$ = −0.63 ± 0.03 kcal/mol from $\tau_{R,1/e}$						
Det–Det120	15.0 ± 2.7	1.5 ± 0.3	45.0 ± 2.4	6.1 ± 0.3	40.1 ± 0.7	34 ± 1	0.0 ± 0.0	17 ± 1	0.999	11 ± 1
Prot–Det120	31.3 ± 0.8	3.3 ± 0.1	38.7 ± 0.7	28 ± 1	28.6 ± 0.9	110 ± 2	1.5 ± 0.0	44 ± 2	0.998	27 ± 1
$\Delta G^o_{SolvEx,Det120Head}$ = −0.58 ± 0.03 kcal/mol from $\langle\tau_R\rangle$				$\Delta G^o_{SolvEx,Det120Head}$ = −0.53 ± 0.06 kcal/mol from $\tau_{R,1/e}$						
Det–Det150	13.3 ± 1.1	0.1 ± 0.3	44.5 ± 1.1	6.4 ± 0.2	41.9 ± 0.6	38 ± 1	0.0 ± 0.0	19 ± 1	0.998	12 ± 1
Prot–Det150	21.0 ± 0.7	0.9 ± 0.1	40.5 ± 0.5	18 ± 1	38.2 ± 0.5	106 ± 1	0.3 ± 0.0	48 ± 1	0.999	30 ± 1
$\Delta G^o_{SolvEx,Det150Head}$ = −0.56 ± 0.02 kcal/mol from $\langle\tau_R\rangle$				$\Delta G^o_{SolvEx,Det150Head}$ = −0.54 ± 0.02 kcal/mol from $\tau_{R,1/e}$						

c

Tail	A_1 (%)	$\tau_{R,1}$ (ns)	A_2 (%)	$\tau_{R,2}$ (ns)	A_3 (%)	$\tau_{R,3}$ (ns)	A_∞ (%)	$\langle\tau_R\rangle$ (ns)	Adj-R^2	$\tau_{R,1/e}$ (ns)
Lip–Lip	25.2 ± 0.5	1.4 ± 0.1	30.8 ± 0.5	19 ± 1	44.1 ± 0.5	81 ± 1	0.0 ± 0.0	42 ± 1	0.999	30 ± 1
Prot–Lip	33.8 ± 0.4	5.5 ± 0.1	44.3 ± 0.5	79 ± 1	17.2 ± 0.5	323 ± 8	1.6 ± 0.0	97 ± 3	0.999	58 ± 1
$\Delta G^o_{SolvEx,LipTail}$ = −0.50 ± 0.02 kcal/mol from $\langle\tau_R\rangle$				$\Delta G^o_{SolvEx,LipTail}$ = −0.39 ± 0.02 kcal/mol from $\tau_{R,1/e}$						
Det–Det120	48.5 ± 0.7	1.6 ± 0.1	29.4 ± 0.8	17 ± 1	21.9 ± 1.0	67 ± 2	0.0 ± 0.0	21 ± 1	0.997	9 ± 1
Prot–Det120	32.1 ± 0.4	3.6 ± 0.1	29.8 ± 0.3	61 ± 1	36.5 ± 0.3	388 ± 3	0.5 ± 0.0	164 ± 2	0.999	87 ± 1
$\Delta G^o_{SolvEx,Det120Tail}$ = −1.22 ± 0.03 kcal/mol from $\langle\tau_R\rangle$				$\Delta G^o_{SolvEx,Det120Tail}$ = −1.34 ± 0.07 kcal/mol from $\tau_{R,1/e}$						
Det–Det150	36.2 ± 1.7	0.9 ± 0.1	28.7 ± 1.7	8.8 ± 1.0	34.6 ± 1.2	55 ± 2	0.5 ± 0.1	22 ± 1	0.998	11 ± 1
Prot–Det150	37.0 ± 0.3	4.6 ± 0.1	36.6 ± 0.3	76 ± 1	23.9 ± 0.4	314 ± 3	0.2 ± 0.0	107 ± 2	0.999	58 ± 1
$\Delta G^o_{SolvEx,Det150Tail}$ = −0.94 ± 0.04 kcal/mol from $\langle\tau_R\rangle$				$\Delta G^o_{SolvEx,Det150Tail}$ = −0.98 ± 0.05 kcal/mol from $\tau_{R,1/e}$						

Figure 1

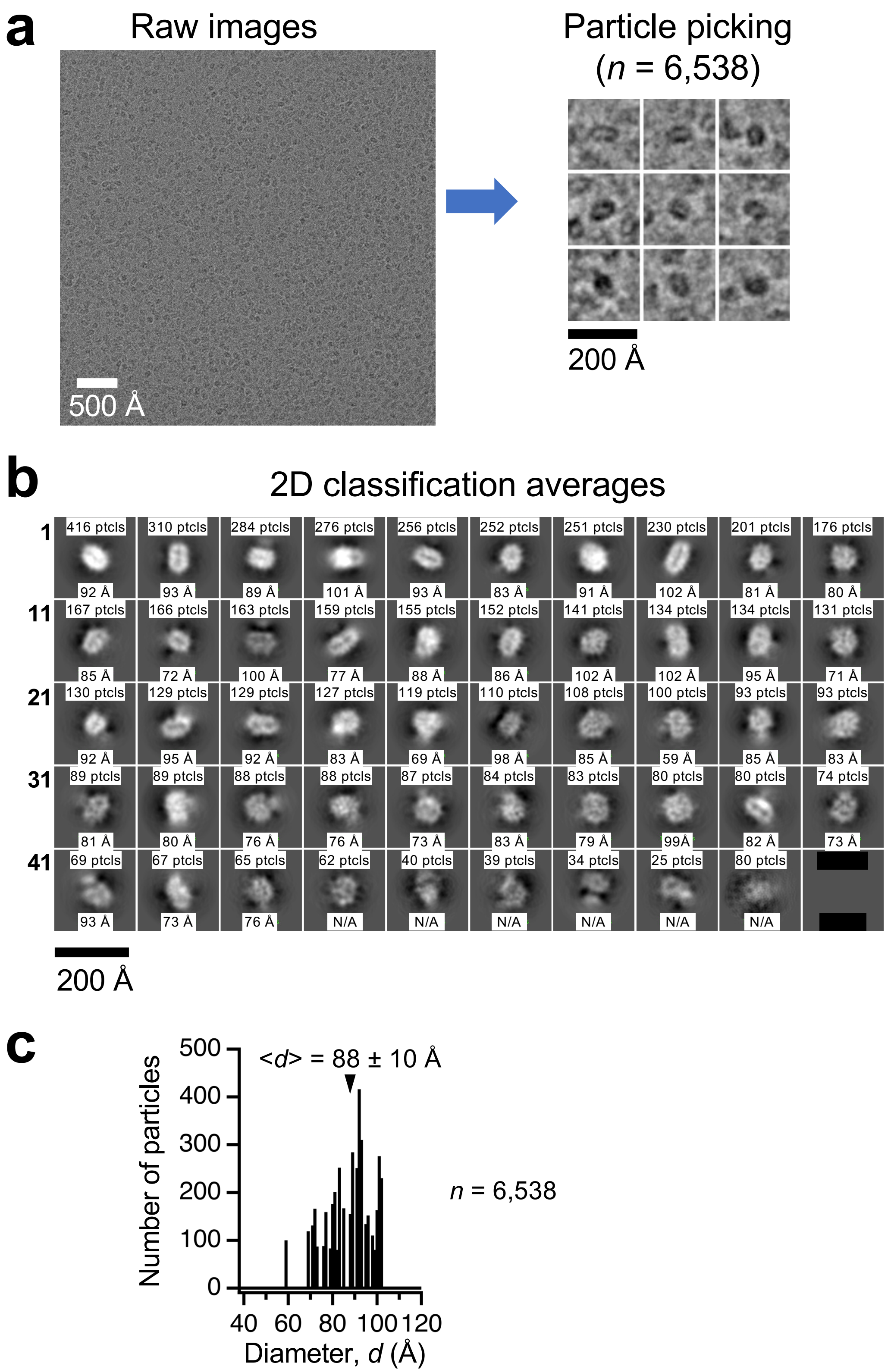

Extended Data Fig.1│ Cryo-EM study of the bicelles used in this study.

(a) (*Left*) The raw images of DMPC:CHAPS bicelles (3.0% w/v and q = 1.5) in 20 mM HEPES (pH 7.5), 40 mM KCl, and 1 mM DTT. (*Right*) Selected particles from the raw images.

(b) 2D-class average images with the number of particles (ptcls) and maximal length for each class average.

(c) The histogram showing the distribution of diameters of bicelles.

Figure 2

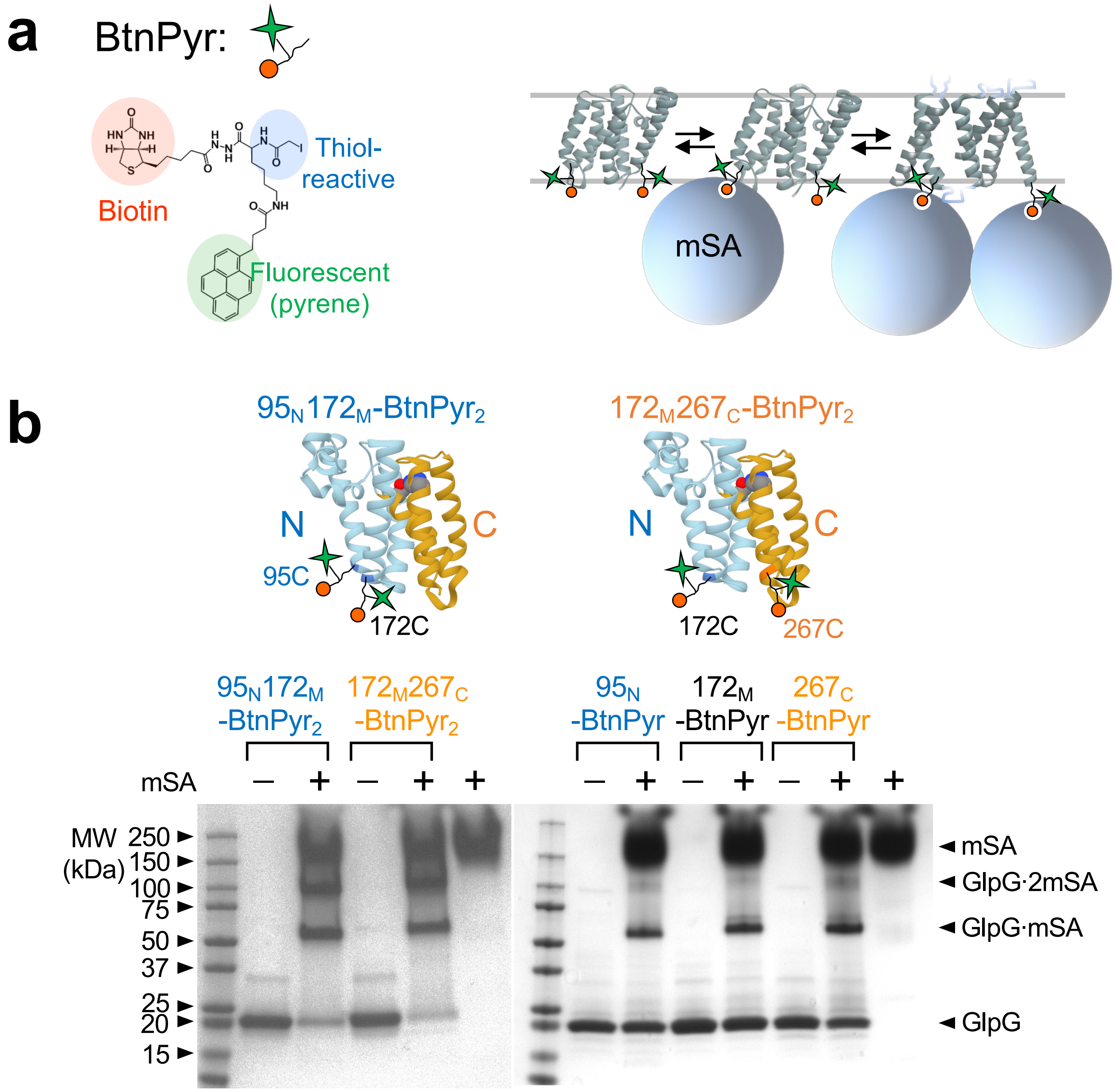

Extended Data Fig. 2│ Cysteine-specific biotinylation of GlpG.

(a) ***(Left)*** Structure of the thiol-reactive biotin derivative possessing a fluorescent pyrenyl group (BtnPyr). (*Right*) The use of BtnPyr to trap denatured GlpG by the addition of excess monovalent streptavidin (mSA).

(b) ***(Left)*** Binding of mSA-WT (the active subunit of mSA is wild type streptavidin with a high biotin-binding affinity, $K_{d,biotin}$ = ~10^{-14} M) to the double-cysteine variants of GlpG (95_N172_M and 172_M267_C) after the biotinylation reaction with BtnPyr. GlpG unbound (GlpG), bound with single mSA (GlpG·mSA), and bound with double mSA (GlpG·2mSA) are separated on SDS-PAGE without sample heating, and then stained by Coomassie Blue G250. The quantification of the band intensities for GlpG, GlpG·mSA, and GlpG·2mSA yields the efficiency of biotinylation, 1.4~1.6 BtnPyr per GlpG. ***(Right)*** Binding of mSA-WT to the single-cysteine variants (95_N, 172_M and 267_C) after the reaction with BtnPyr. The major reaction products are GlpG (unbound) and GlpG·mSA with a minor fraction of GlpG·2mSA (over-labeled). Tetrameric mSA-WT (52 kD) migrates as a large species (>150 kD) probably due to an abnormal number of SDS molecules bound to a tetrameric mSA-WT molecule under the condition of no sample heating.

Figure 3

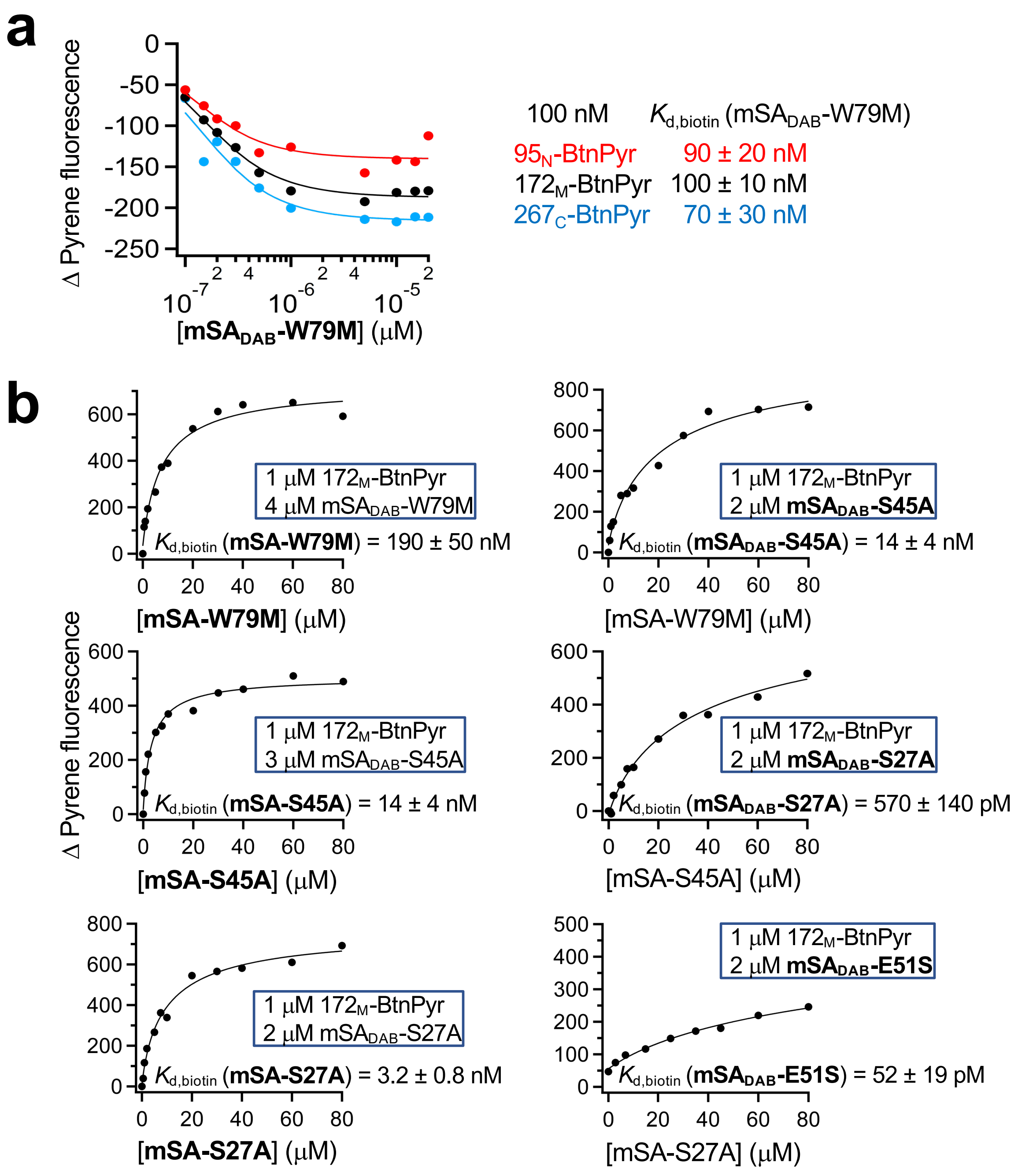

Extended Data Fig. 3│ Determination of the binding affinities ($K_{d,biotin}$) of mSA variants to biotin labels (BtnPyr) on GlpG using Förster resonance energy transfer (FRET) in bicelles.

(a) Binding isotherms between a weak binding variant mSA-W79M with the dabcyl quencher (mSA_{DAB}-W79M) and the GlpG variants with a single biotin label at three different positions. The data were fitted to **Methods Eq. 5** to determine $K_{d,biotin}$. $K_{d,biotin}$'s at three biotin sites are similar.

(b) Competition assays to determine $K_{d,biotin}$ between high-affinity mSA variants and a single-biotin variant label at G172C on GlpG. (*Top left*) The complex of 172_M-BtnPyr and mSA_{DAB}-W79M with known $K_{d,biotin}$ was incubated at an increasing concentration of mSA-W79M (no dabcyl label, unknown $K_{d,biotin}$). The replacement of mSA_{DAB}-W79M by mSA-W79M in the complex leads to an increase in pyrene fluorescence, which was fitted to **Methods Eq. 6** to determine the $K_{d,biotin}$ of mSA-W79M. (*Top right)* The $K_{d,biotin}$ of the next higher affinity variant mSA_{DAB}-S45A was determined using mSA-W79M with known $K_{d,biotin}$ obtained from the preceding plot. Using the same strategy, $K_{d,biotin}$'s of mSA_{DAB}-S27A and mSA_{DAB}-E51S were determined consecutively.

Figure 4

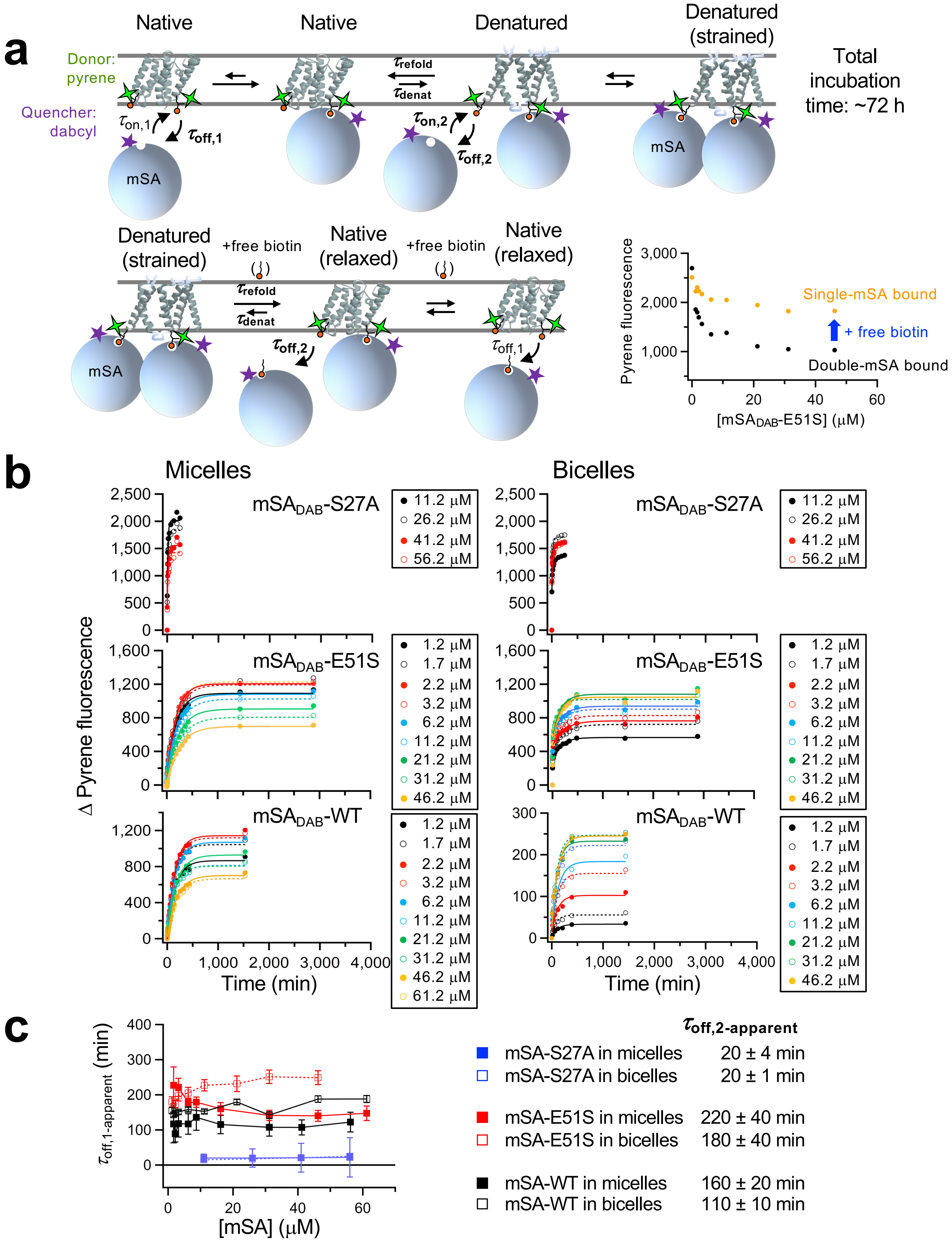

Extended Data Fig. 4│ Determining the dissociation time constant ($\tau_{off,1\text{-}apparent}$) of mSA variant from sterically denatured GlpG. Figure legends are shown in the next page.

Extended Data Fig. 4│ Determining the dissociation time constant ($\tau_{off,1\text{-}apparent}$) of mSA variant from sterically denatured GlpG in bicelles.

(a) Here, we compared (*Top*) the total incubation time of the steric trapping reaction (~72 h) to (*Bottom left*) the time scales of the dissociation ($\tau_{off,2}$) time constants of the second mSA molecule from sterically denatured GlpG. Here, we evaluated the dissociation time constant of the mSA molecule ($\tau_{off,2}$) that induces the refolding of sterically denatured GlpG. The on-rates of mSA molecules (k_{on} = ~10^6 to 10^7 $s^{-1}M^{-1}$) to transiently denatured GlpG are expected to be larger than the off-rates of the mSA-biotin complex (Srisa-Art et al. *Anal Chem* 2008 80, 7063-7). If the dissociation ($\tau_{off,2}$) occurs in the faster time scales than the total incubation time, the observed binding isotherms for the second mSA binding (*bottom right*, black circles) can be regarded truly equilibrated, and thus $\Delta G^o_{N\text{-}D}$ of GlpG can be determined from the attenuated second mSA binding. Detailed description of each plot is shown below.

(*Top*) The equilibrium reaction scheme of steric trapping with the time constants of each reaction step. (*Bottom left*) The schematic description of measuring the time constant of a first dissociation of mSA ($\tau_{off,2}$) from sterically denatured GlpG. The double-biotin variant of GlpG, 172_M267_C-BtnPyr$_2$ (1 μM), is first denatured at an excess concentration of mSA$_{DAB}$ variants. In this initial state, denatured GlpG is strained by the steric hindrance between doubly-bound mSA$_{DAB}$ molecules, and pyrene fluorescence from the BtnPyr labels on GlpG are quenched by the dabcyl quencher on mSA$_{DAB}$. The addition of excess free biotin (2 mM) induces the dissociation of one bound mSA$_{DAB}$ molecule to relieve the strain, and the pyrene fluorescence increases by dequenching. (*Bottom right*) An example of the binding isotherm between mSA and GlpG (denaturation, black circles) and fluorescence dequenching upon addition of free biotin (refolding, orange circles).

(b) The assay result. Upon addition of excess free biotin, the dissociation kinetics of mSA$_{DAB}$ variants bound to 172_M267_C-BtnPyr$_2$ were measured at an increasing concentration of mSA$_{DAB}$. The data was fitted to a single exponential function under the assumption of the pseudo-first order reaction.

(c) The pseudo-first order dissociation time constants ($\tau_{off,2\text{-}apparent}$) of mSA$_{DAB}$ variants are independent of the concentration of mSA$_{DAB}$. The slower time constants of mSA$_{DAB}$ are in the range of 2 h to 4 h, shorter than the total incubation time (~72 h). Thus, $\Delta G^o_{N\text{-}D}$ of GlpG can be determined from the "equilibrated" attenuated second mSA binding (**Fig. 1e** and **Extended Data Fig. 9**).

Figure 5

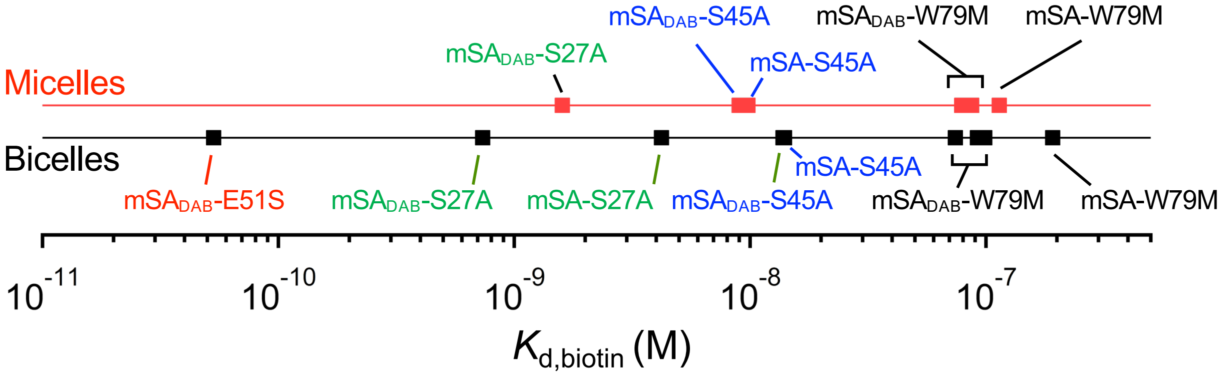

Extended Data Fig. 5│ The intrinsic binding affinities ($K_{d,biotin}$) of mSA variants to the biotin label on GlpG (BtnPyr) in micelles and bilayers.

The $K_{d,biotin}$'s for mSA_{DAB}-W79M (dabcyl-labeled), mSA-W79M (unlabeled), mSA_{DAB}-S45A, mSA-S45A, mSA_{DAB}-S27A, and mSA-S27A were determined in DDM micelles, previously (Guo et al. 2016 *Nat Chem Biol* 12, 353). Here, the $K_{d,biotin}$'s of mSA_{DAB}-W79M, mSA-W79M, mSA_{DAB}-S45A, mSA-S45A, mSA_{DAB}-S27A, mSA-S27A, and mSA_{DAB}-E51S were determined in 2.0% (w/v) DMPC:CHAPS bicelles (molar ratio = 1.5:1). Overall, for a given variant, the discrepancy between the $K_{d,biotin}$'s in micelles and those in bicelles is less than two folds, which translates into the changes of ~0.4 kcal/mol in $\Delta G^{o}_{N\text{-}D}$.

Figure 6

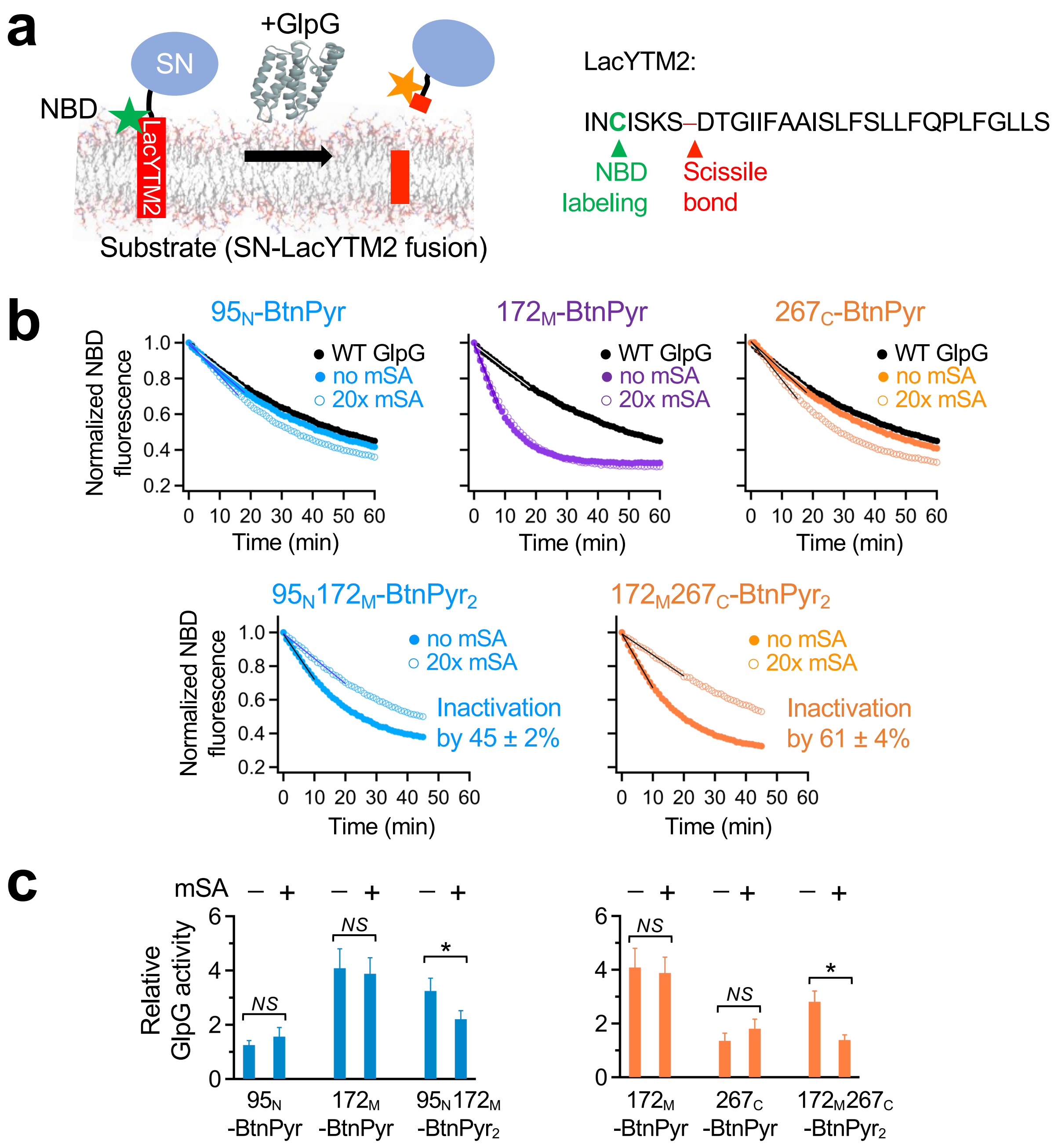

Extended Data Fig. 6│ Activity assays to measure GlpG denaturation induced by steric trapping.

(a) (*Left*) Schematic description of an assay to measure the proteolytic activity of GlpG using the transmembrane (TM) model substrate SN-LYTM2 by GlpG (SN: staphylococcal nuclease fusion; LYTM2: the second TM segment of *E. coli* lactose permease). (*Right*) SN-LYTM2 is labeled with the environment-sensitive fluorophore NBD on the the five-residue upstream (Cys) of the scissile bond. The cleavage of LYTM2 induces the transfer of NBD from the hydrophobic bicelles to the aqueous phase. The transfer induces the decrease in NBD fluorescence.

(b–c) The effects of mSA binding to the single- and double-biotin variants of GlpG on activity in bicelles. The activity is defined as the initial slope of time-dependent decrease of NBD fluorescence. The inactivation (*i.e.*, denaturation) occur only upon saturated binding of mSA to the double biotin variants, not upon binding to the individual single-biotin variants. The *p*-values for the student *t*-test are shown (NS: $p>0.05$; *: $p<0.05$).

Figure. 7

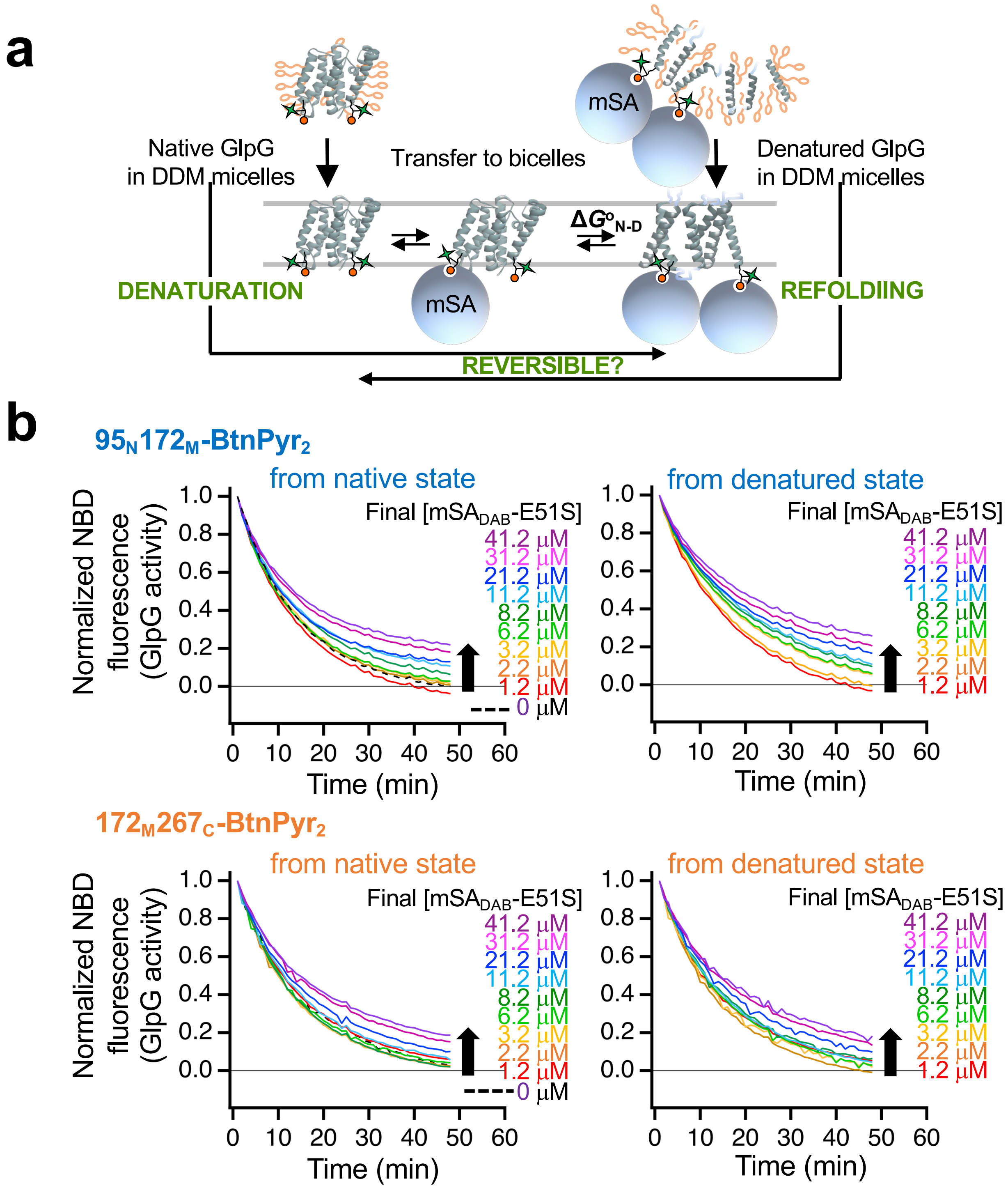

Extended Data Fig. 7│ Testing the reversibility of GlpG folding in bicelles monitored by GlpG activity (This data supports the results in **Fig. 1c**).

(a) The strategy. Native or sterically denatured GlpG in micelles is transferred to bicelles at an increasing concentration of mSA_{DAB}-E51S labeled with dabcyl quencher. After incubation for 72 h, the activity of GlpG was measured as a folding indicator at each [mSA_{DAB}-E51S].

(b) The assay results for 1 μM GlpG in 3% (w/v) DMPC/CHAPS bicelles (q = 1.5) at room temperature. Quenching of NBD fluorescence was monitored upon cleavage of the model substrate LYTM2 labeled with NBD (**Extended Data Fig. 3a**). As the [mSA_{DAB}-E51S] increased (black block arrows), the activity decreased (*i.e.*, the decrease in the initial slope), indicating that the fraction of denatured GlpG increased. For each double-biotin variant of GlpG, the degree of inactivation was similar at a given [mSA_{DAB}-E51S] regardless of the initial state in micelles before transfer to bicelles.

Figure 8

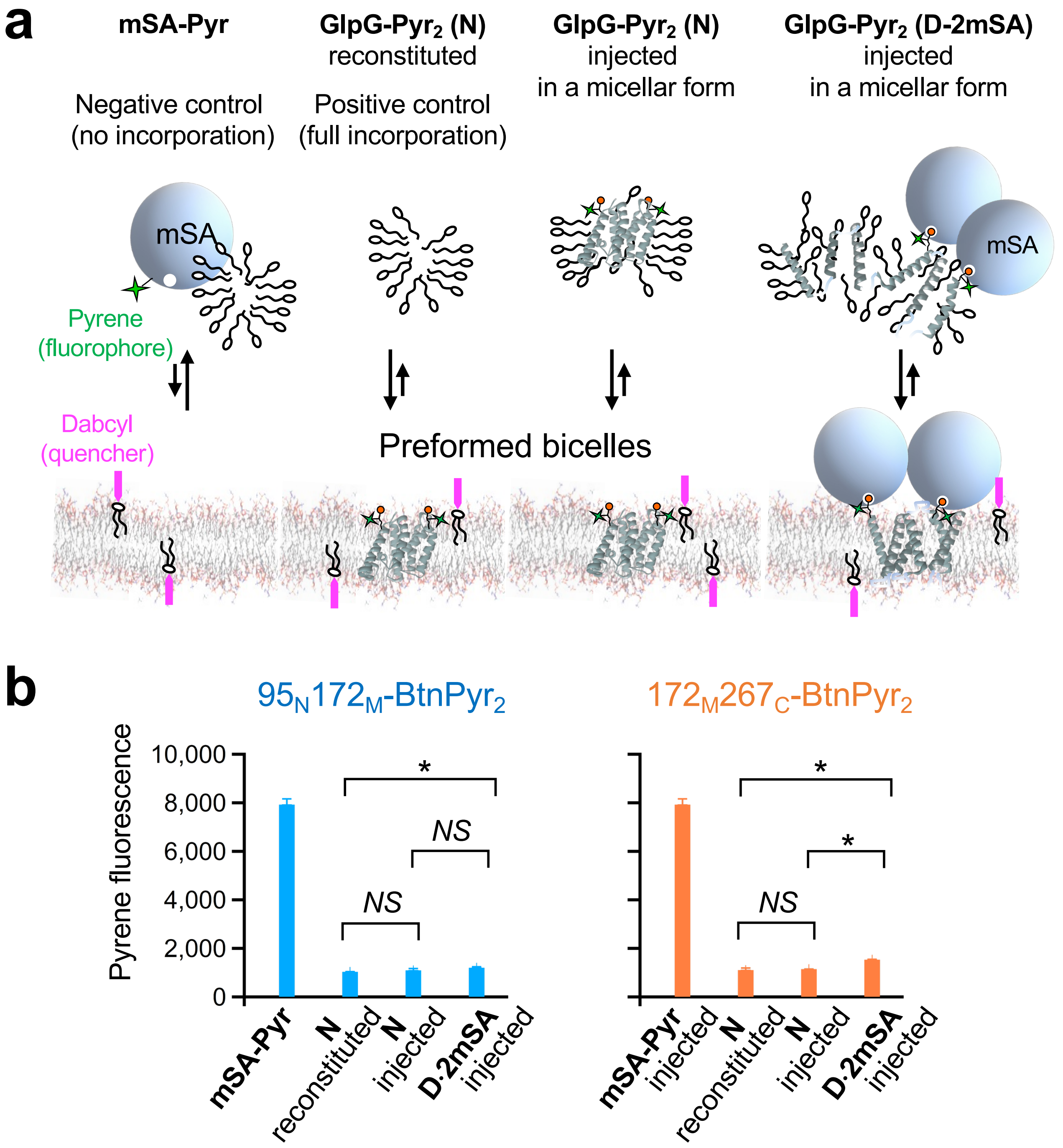

Extended Figure 8│ Incorporation of native and sterically denatured GlpG into bicelles.

(a) Schematic description of a fluorescence quenching assay to measure the transfer of native (N: the folded double-biotin variants, GlpG-BtnPyr$_2$) and sterically denatured GlpG (D·2mSA) from the micellar to the bicellar phase by direct injection. The preformed bicelles contain dabcyl (quencher)-labeled lipid (the molar ratio, DMPC:dabcyl-DOPE = 199:1). Pyrene-labeled mSA (mSA-Y83C-Pyr), which is soluble in water, was used as a negative control (*i.e.*, no incorporation). Native GlpG-BtnPyr$_2$, which was first reconstituted in DMPC liposomes and then solubilized by CHAPS to form bicelles, was used as a positive control (*i.e.*, full incorporation).

(b) The result of the assay. Incorporation of GlpG-BtnPyr$_2$ to bicelles induced quenching of pyrene fluorescence. Error bars denote ± SEM. (n = 3). The p-values for the student t-test are shown (*NS*: $p>0.05$; *: $p<0.05$).

Figure 9

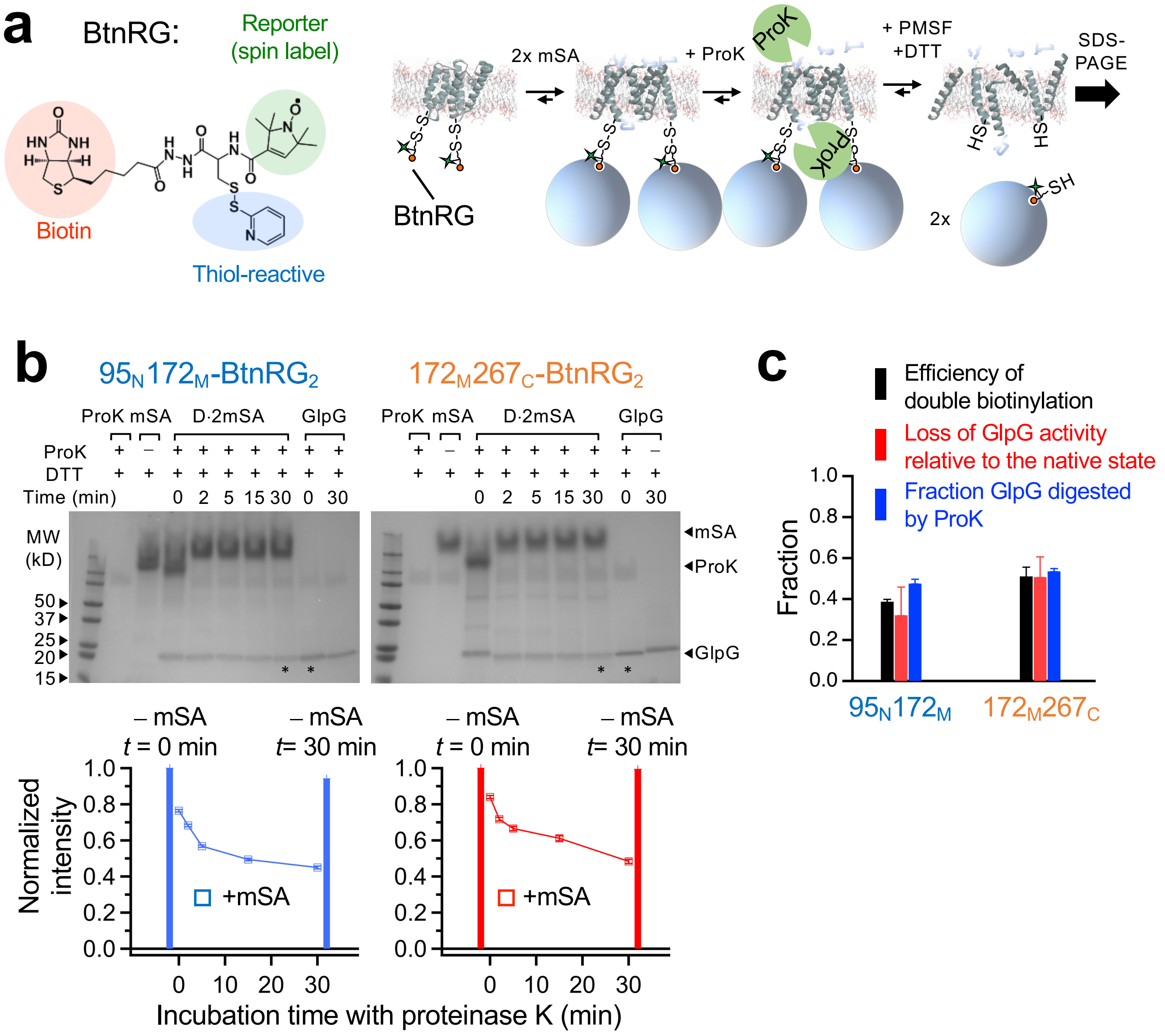

Extended Data Fig. 9│ Denaturation of GlpG induced by steric trapping.

(a) (*Left*) Structure of reversible thiol-reactive biotin derivative (BtnRG-TP). Thiopyridine group reacts with the thiol group in a cysteine residue to form a disulfide linkage. (*Right*) Strategy to detect sterically denatured GlpG by proteolysis. GlpG labeled with BtnRG is denatured by steric trapping. Denatured GlpG reacts with ProK for various incubation times. At each incubtation time, the protease inhibitor PMSF and the reducing agent DTT are added to inactivate ProK and to break the linkage between GlpG and the biotin label bound with mSA, respectively. The final products are analyzed by SDS-PAGE.

(b) (*Top*) Selective digestion of sterically denatured GlpG monitored by SDS-PAGE as a function of incubation time with ProK. (*Bottom*) The band intensities of GlpG on the SDS-PAGE gel analyzed by the ImageJ program. As controls, the intensities of GlpG without mSA and with ProK at time 0 and 30 min are shown, respectively.

(c) Correlation between the efficiency of double biotinylation (**Extended Data Fig. 1**), activity loss induced by steric trapping and digestion by ProK (**Extended Data Figs. 3** and **4b**). The incomplete digestion in the presence of excess mSA is due to the incomplete double-biotin labeling of GlpG.

Figure. 10

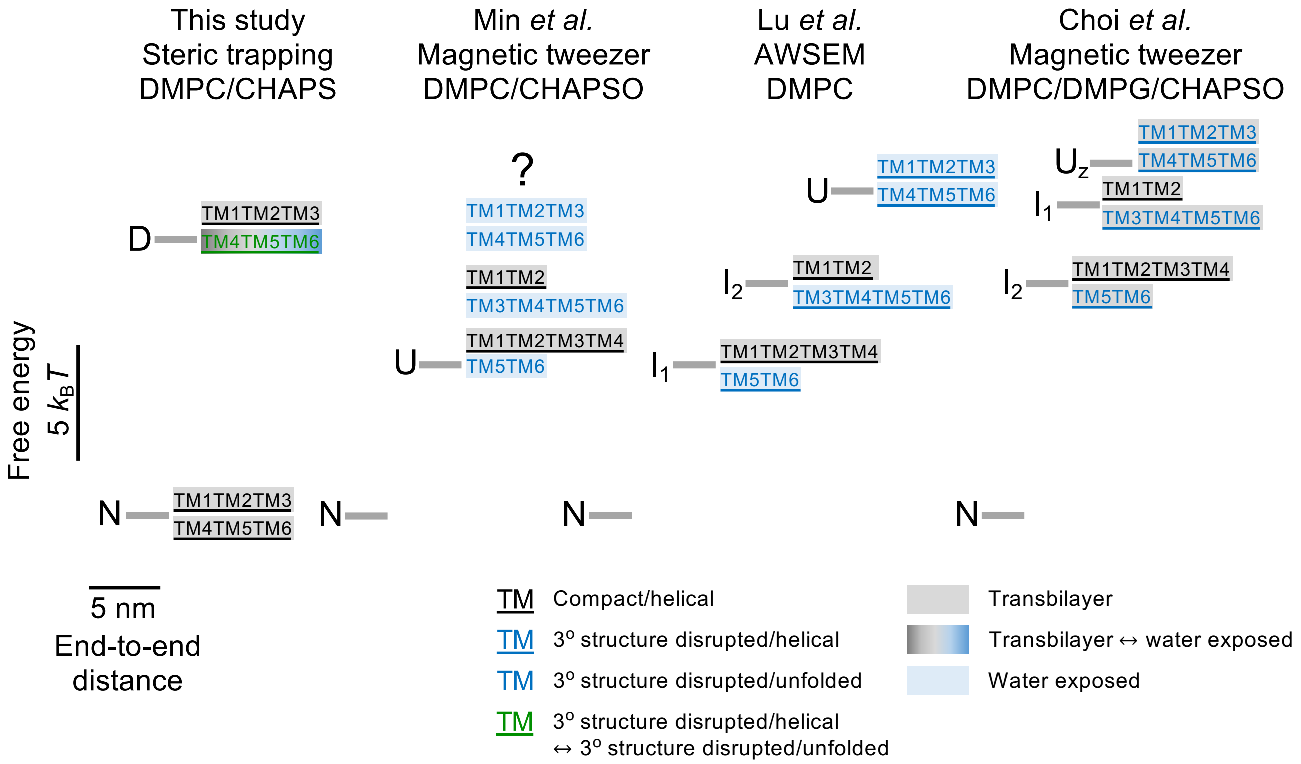

Extended Data Fig. 10│ Comparison of the free energy landscapes of GlpG folding in the bilayer measured with various methods.

The free energy level (in k_BT) and the degree of compactness (the end-to-end distance between the N- and C-termini) of each state are shown relative to the native state ("N"). The conformation of each state is also shown regarding whether the tertiary interaction is maintained ("compact" vs "3° structure disrupted"), the secondary structure is maintained ("helical" vs "unfolded"), or each structural element is buried in the membrane ("transbilayer" vs "exposed to water"). "D": denatured state; "I": intermediate state; "U": unfolded state.

The stability of GlpG that we determined directly under native condition ($\Delta G^o_{N\text{-}D}$ = –12k_BT, "This study") in bicelles is much larger than that from the single-molecule magnetic tweezer study in the same neutral bicelles (–6.5k_BT, "Min *et al.*").[54] In the latter, $\Delta G^o_{N\text{-}D}$ was obtained by extrapolating the unfolding and refolding rates measured in the two distinct force ranges (12–30 pN and 2–7 pN, respectively) to zero force. At the higher force, GlpG unfolds *via* a single cooperative step or multiple steps with one or two intermediates to the fully stretched coil.[54] At the lower force, the conformation of the starting unfolded state prior to refolding is not defined ("?"). A simulation study("Lu *et al.* AWSEM") predicts that the unfolded state at low force is I_1 (TM1–TM4 folded).[55] Notably, our $\Delta G^o_{N\text{-}D}$ is similar to the free energy difference between the native state and I_2 (TM1–TM2 folded) from the same simulation (–10k_BT)[55] as well as that between the native state and I_1 (TM1–TM2 folded) from the more recent tweezer study in the negatively charged bicelles (–13k_BT)[56] at low force ("Choi *et al.*"). We note that the end-to-end distances for "N" and "D" states in "This study" were taken from our previous work (Gaffney et al.) measured in the negatively charged bicelles (DMPC/DMPG/CHAPS) using DEER.

Previously, we have shown that while both N- (TM1–TM3) and C- (TM4–TM6) subdomains in sterically denatured GlpG expand relative to those in the native state in bicelles, N-subdomain denatures close to the collapse limit with C-subdomain close to the full-expansion limit, resembling the conformation expected from I_2 by "Lu *et al.*" and from I_1 by "Choi *et al.*"[42] *Thus, we reason that the stability discrepancy likely stem from the different conformation of the denatured state in the steric trapping ("This study") and magnetic tweezer ("Min et al.") studies.*

Figure 11

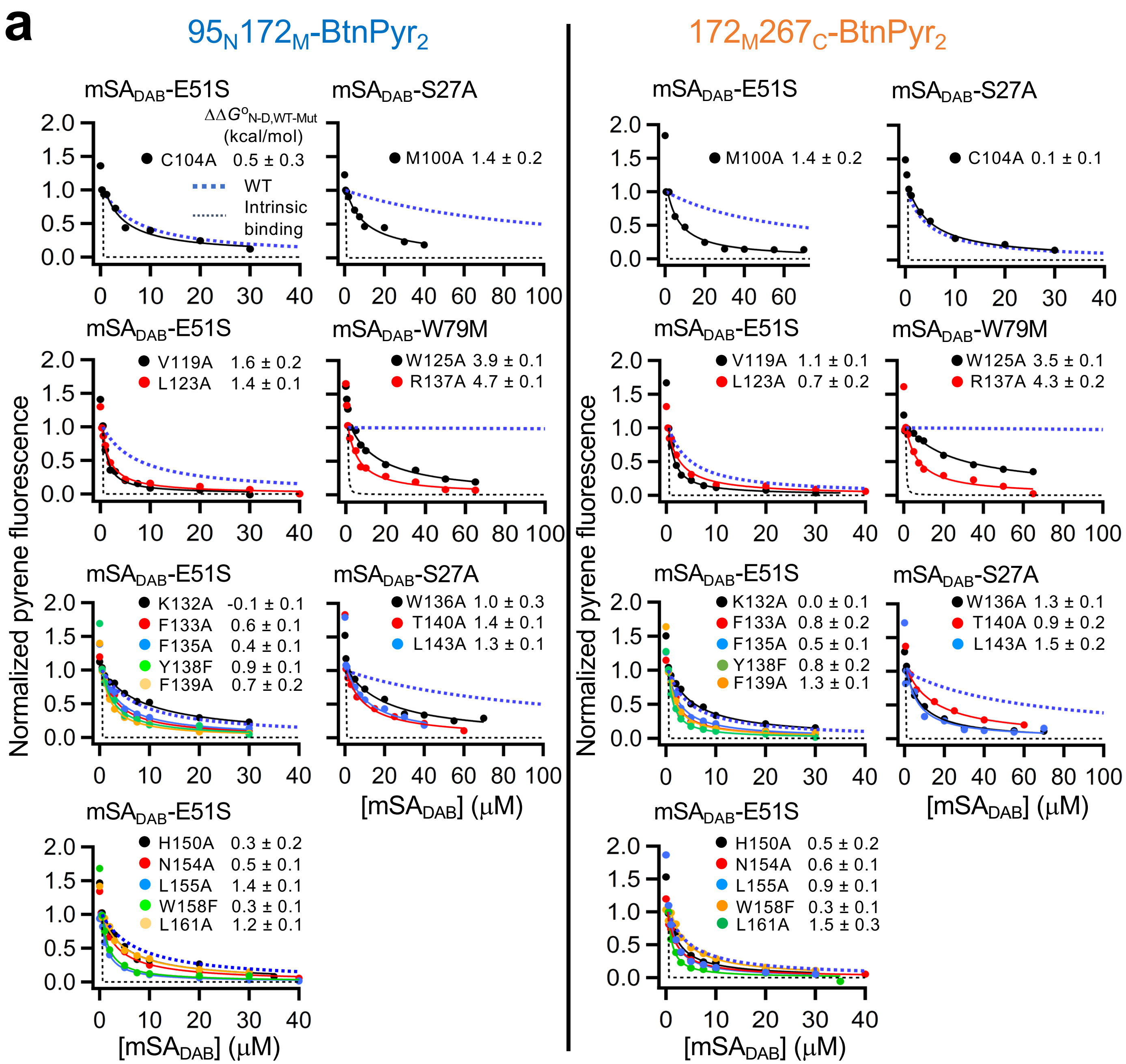

Extended Data Fig. 11│ Binding isotherms between the double-biotin variant of GlpG (95_N172_M-BtnPyr$_2$ or 172_M267_C-BtnPyr$_2$) and monovalent streptavidin (mSA) to determine the thermodynamic stability of GlpG in DMPC/CHAPS bicelles using steric trapping. Binding was measured by quenching of pyrene fluorescence from the BtnPyr labels on GlpG, which was induced by the dabcyl quencher conjugated to mSA (mSA$_{DAB}$). The first mSA binds either biotin label with an intrinsic binding affinity (black dashed lines). Binding of the second mSA is attenuated depending on the stability of GlpG ($\Delta G^o_{N\text{-}D}$), which was obtained by fitting the attenuated second binding phase to **Methods Eq.'s 1–2**. In each plot, the fluorescence intensity was normalized to the intensity change of the second binding phase and the WT data is shown. The difference stability between WT and mutant ($\Delta\Delta G^o_{N\text{-}D,WT\text{-}Mut} = \Delta G^o_{N\text{-}D,WT} - \Delta G^o_{N\text{-}D,Mut}$) is shown. An mSA variant was chosen using the criteria that the attenuated second binding phase was observed over the range of 1 μM to 40-80 μM. *The more attenuated second binding indicates the higher stability (i.e., the larger $\Delta G^o_{N\text{-}D}$).*

a: Binding isotherms for the variants bearing a mutation on the segments TM1, L1 and TM2 of GlpG.

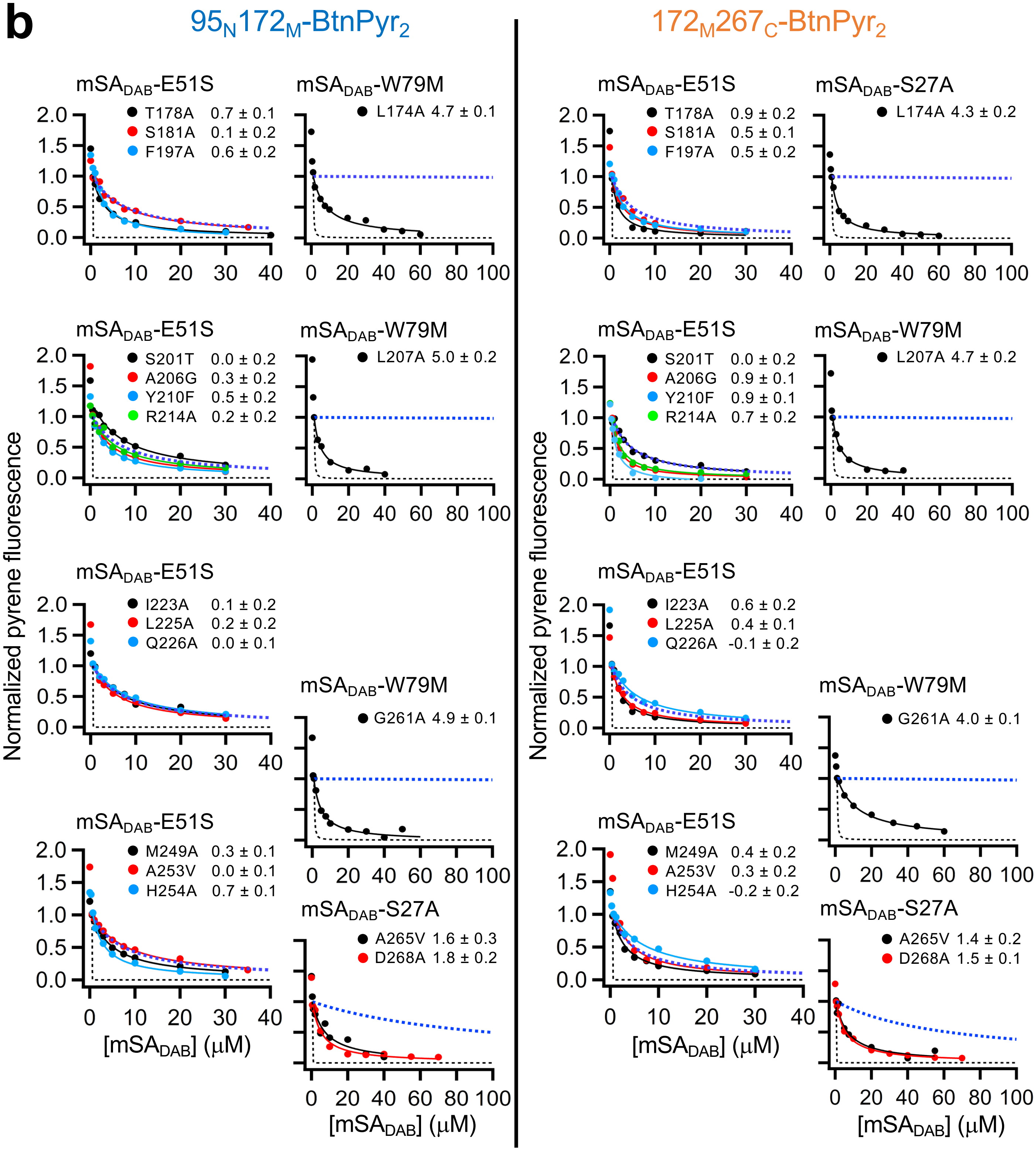

Continued-Extended Data Fig. 11│ Binding isotherms between the double-biotin variant of GlpG (95_N172_M-BtnPyr$_2$ or 172_M267_C-BtnPyr$_2$) and monovalent streptavidin (mSA) to determine the thermodynamic stability of GlpG in DMPC/CHAPS bicelles.

b: Binding isotherms for the variants bearing a mutation on the segments TM3, TM4, TM5 and TM6 of GlpG.

Figure 12

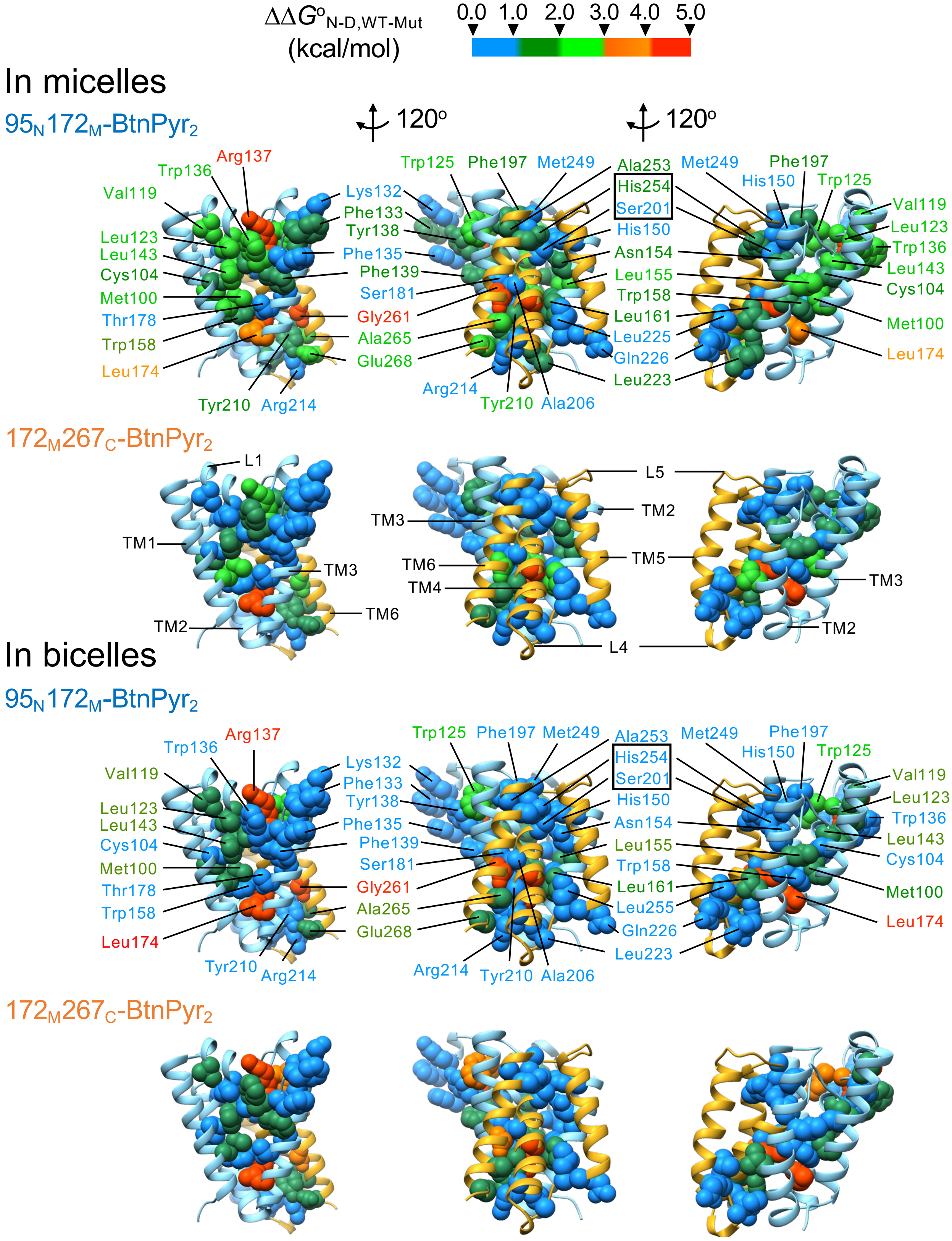

Extended Data Fig. 12│ Mapping of the mutation-induced stability changes onto GlpG structure.

The degree of stability change (**$\Delta\Delta G^{o}_{N\text{-}D,WT\text{-}Mut}$ = $\Delta G^{o}_{N\text{-}D,WT}$ – $\Delta\Delta G^{o}_{N\text{-}D,Mut}$**) measured at N and C subdomains ($95_{N}172_{M}$-BtnPyr$_2$ and $172_{M}267_{C}$-BtnPyr$_2$, respectively) in micelles and bicelles were color-coded (*Top*) as a heat map on the structure (PDB code: 3B45).

Figure 13

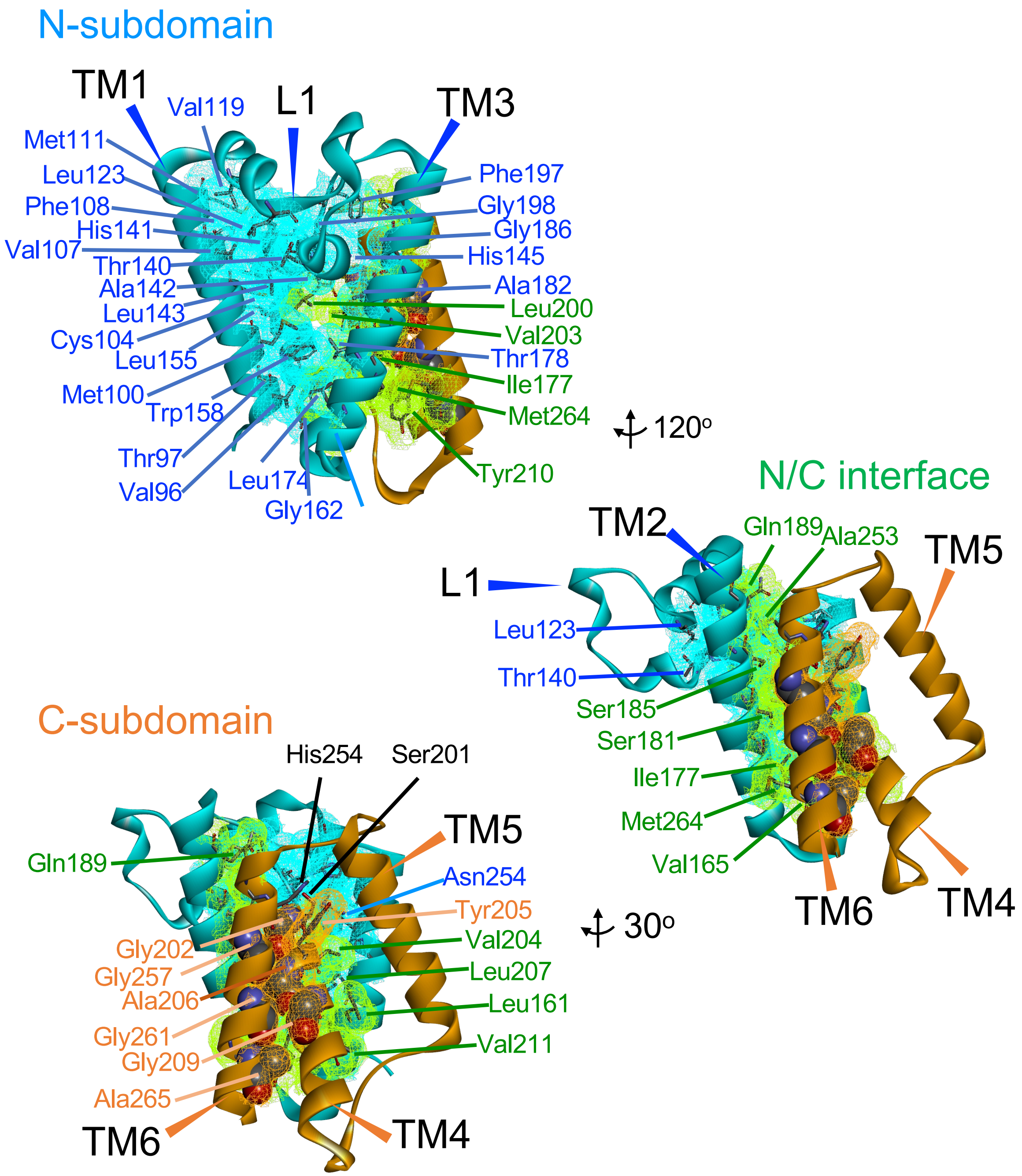

Extended Data Fig. 13│ Residue packing in N-, C-subdomains, and subdomain interface.

The residues that participate in the packing of N- (blue, *top*), C- (orange, *bottom*) subdomain, and subdomain interface (green, *middle*) in the structure of GlpG (PDB code: 3B45). The interior of N-subdomain and the subdomain interface are mainly formed by the extensive knob-into-hole type vdW packing of the large (Thr, Val, Leu, Met, Phe and Trp) and small (Gly, Ala and Ser) nonpolar residues. C-subdomain is primarily mediated by the face-to-face backbone contact between TM4 and TM6 through the Gly-zipper motifs (GlyxxxGlyxxxGly: x is any residue and Gly can be replaced by Ala or Ser). The catalytic dyad Ser201-His254 (black, bottom) is mounted on the helix-helix interface formed by TM4 and TM6.

Figure 14

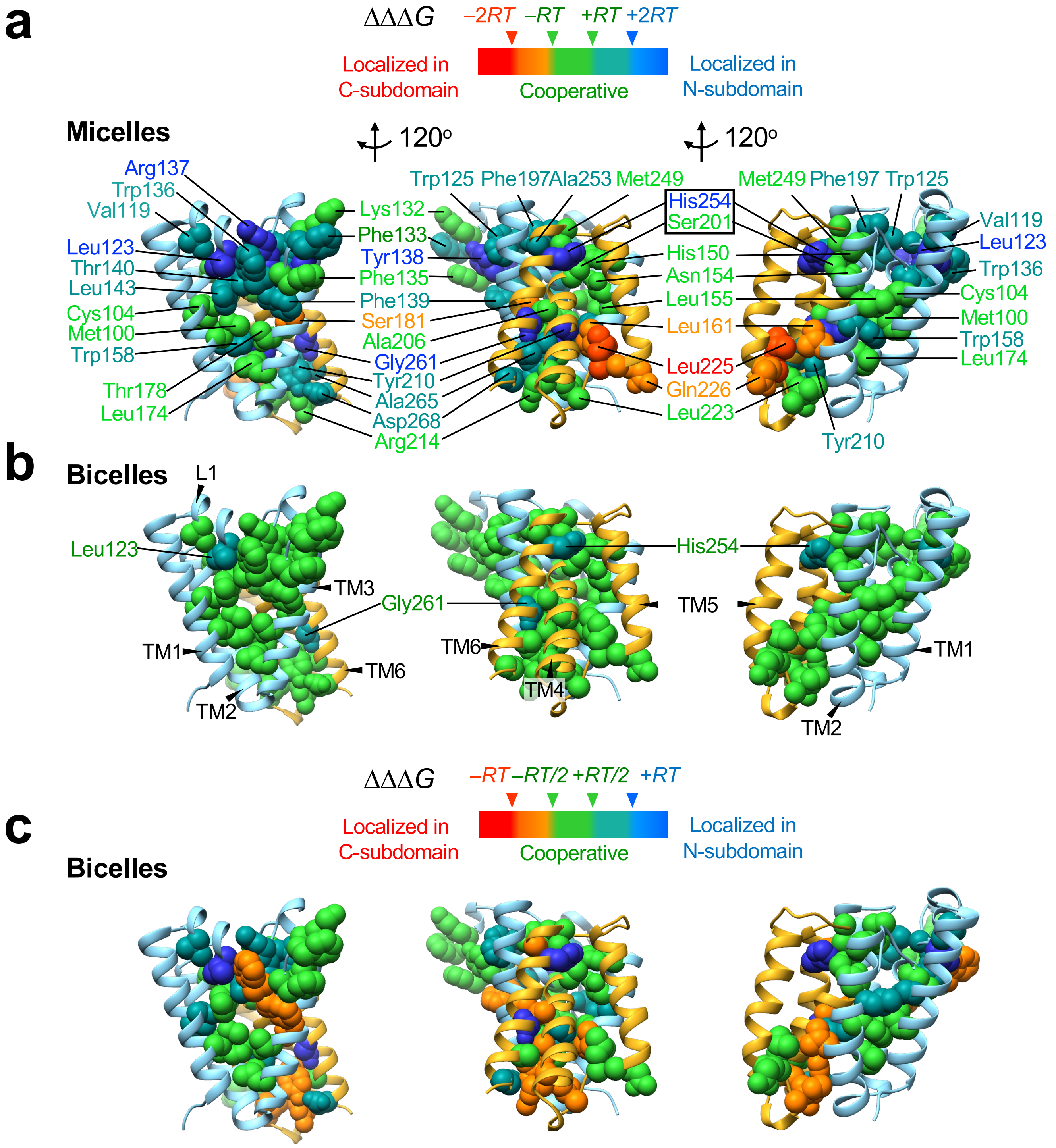

Extended Data Fig. 14│ The features of cooperativity profiles in lipid bilayers still preserve those in micelles, but to a less extent.

(a–b) Comparison of cooperativity profiles between micells (***Top***) and bilayers (***Bottom***) on the basis of the cut-off values, –2*RT*, –*RT*, +*RT*, and +2*RT* (i.e., the *RT* scale).

(c) Cooperativity profiles in bilayers on the basis of smaller cut-off values, –*RT*, –1/2*RT*, +1/2*RT*, and +*RT* (i.e., the 1/2*RT* scale). Overall, except for several residues which display distinctively different profiles (Phe135, Phe136, Ala203, Ala206, Leu225, Gln226, and Arg214), the reconstructed profiles using the 1/2*RT* scale in bilayers has an overall similarity to those using the *RT* scale in micelles. The preserved features include: the cooperative packing core (formed by TM1, TM2 and TM3), the cooperative cluster in the active site (Ser201, His150 and Asn154), the localized cluster in L1, and the overpropagated cluster at the TM4-TM6 interface.

Figure 15

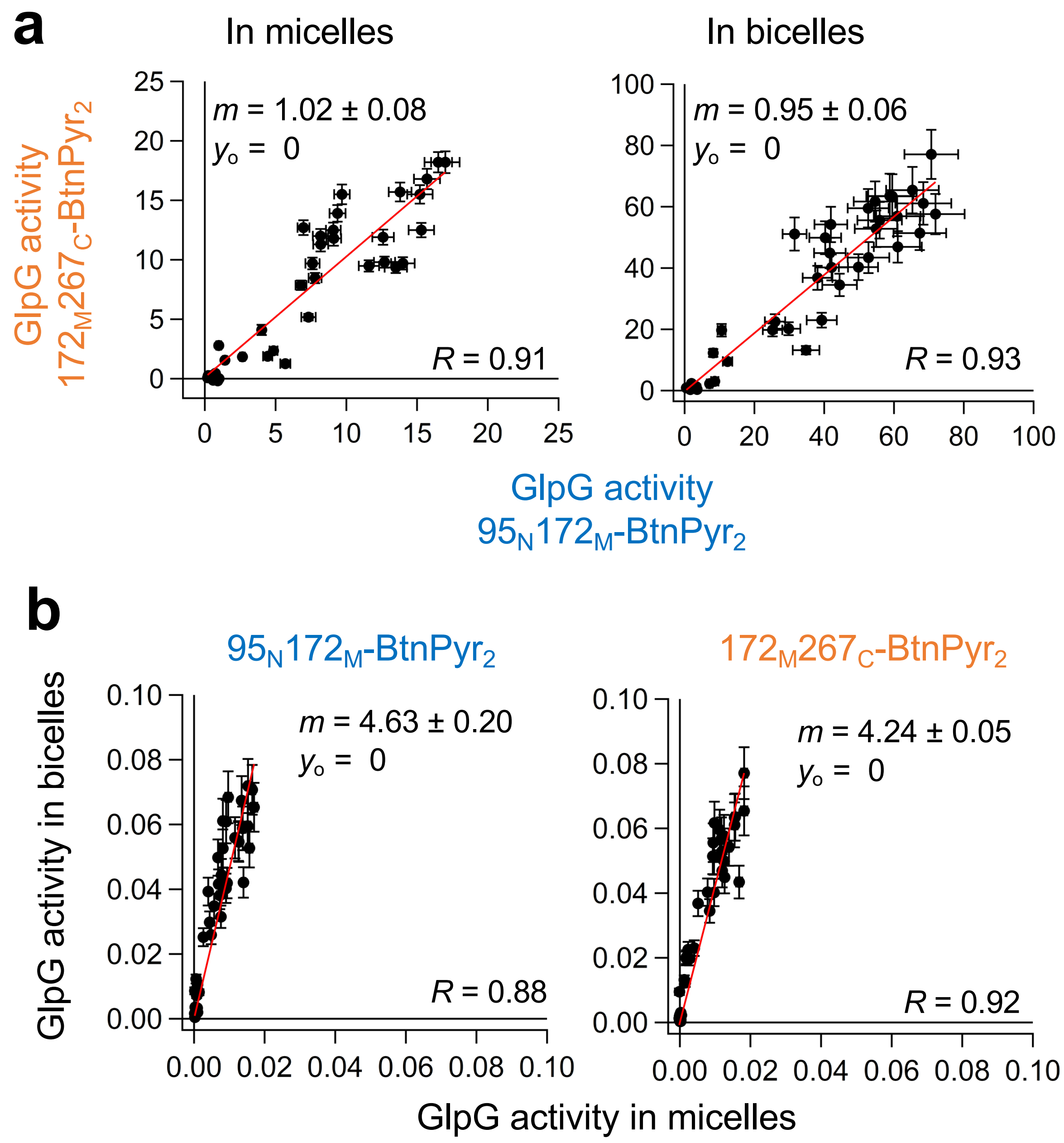

Extended Data Fig. 15│ The effects of the location of the biotin pair and the hydrophobic environment on the proteolytic activity of GlpG variants.

(a) The effect of the location of the biotin pair (172/267$_C$-BtnPyr$_2$ *vs* 172/267$_C$-BtnPyr$_2$) on GlpG activity for the TM substrate LYTM2 in micelles and bilayers. In both environments, the slopes (the activities of 172/267$_C$-BtnPyr$_2$ *vs* 172/267$_C$-BtnPyr$_2$) are close to the unity, indicating that the location of the biotin pair does not affect the mutational impacts on activity.

(b) The effect of the hydrophobic environment (micelles vs bicelles) on GlpG activity for the TM substrate LYTM2. All activity values correspond to the fractional substrate turnover rate (min^{-1}) out of the initial substrate concentration (10 μM) in DDM micelles (5 mM) or DMPC:CHAPS (2% w/v, *q* = 1.5) bicelles as measured by NBD fluorescence (**Extended Data Figs. 3a-b**). Errors denote SEM (*n* = 3).

Figure 16

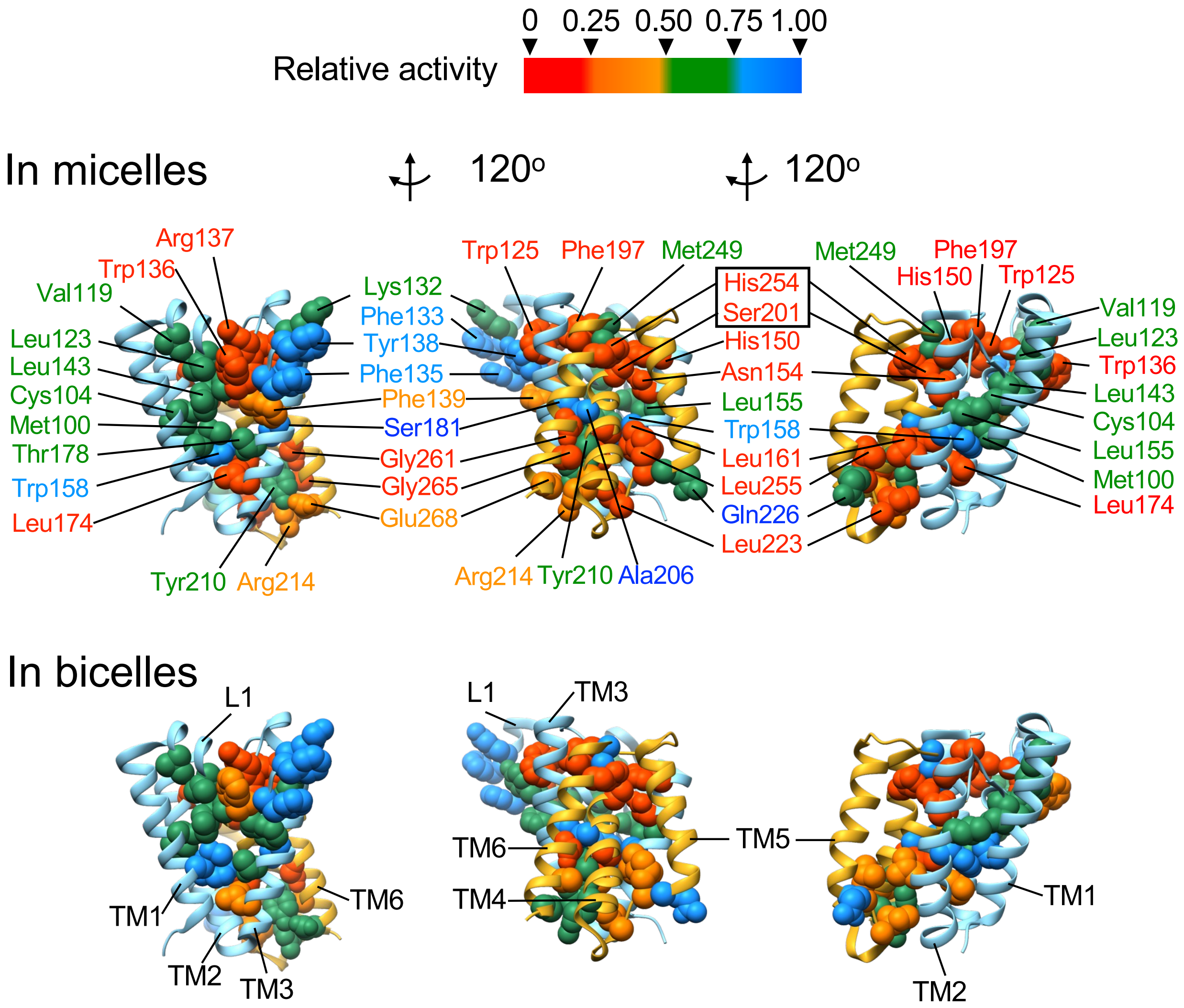

Extended Data Fig. 16│ Mapping of the mutation-induced activity changes onto the structure of GlpG.

For each mutation, the relative activities measured in the backgrounds of 95_N172_M-BtnPyr$_2$ and 172_M267_C-BtnPyr$_2$ (**Extended Data Tables 1 and 2**) were averaged and color-coded in the heat map on the structure (PDB code: 3B45). Overall, the inactivating mutations are distributed at the TM4/TM6 interface harboring the catalytic dyad (Ser201-His254 marked with a rectangular box), in the L1 loop (Arg136 and Trp137, Wang & Ha 2007 *J Mol Biol* 374, 1104), and the substrate binding site at the TM2/TM5 interface. The activity is slightly more tolerant to mutation in bicelles.

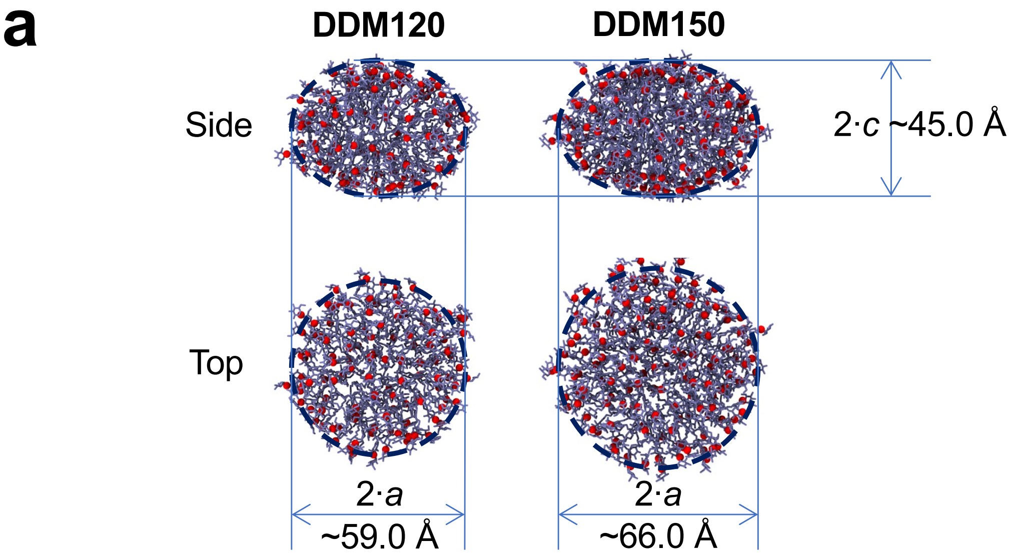

b

Oblate structure	DDM120	DDM150	SAXS ($N_{A,DDM}$ = 135-149)
Radius, *a*	29.5 ± 0.8 Å	32.9 ± 0.8 Å	33.5 ± 0.3 Å
Radius, *c*	22.6 ± 1.1 Å	22.5 ± 0.9 Å	21.3 ± 0.3 Å
Oblateness	0.23 ± 0.06	0.31 ± 0.04	0.36 ± 0.01
Area per DDM	77.3 ± 1.6 Å^2	72.1 ± 1.7 Å^2	76.3 ± 4.0 Å^2

Extended Data Fig. 17│ Modelling of the micellar systems for MD simulation.

(a) Two micellar systems in this study, one with 120 DDM molecules (DDM120) and the other with 150 molecules (DDM150). The shapes of both micellar systems were oblate with the axial dimension (2x*c*), which remained constant at *c* = ~22.5 Å.

The shape of micelles was assessed for DDM120 and DDM150 without protein to understand overall packing. The structure of a DDM micelle was approximated by a spheroid as below:

$$\frac{x^2}{a^2}+\frac{y^2}{a^2}+\frac{z^2}{c^2}=1$$

, where *x*, *y*, and *z* are the cartesian coordinates in the 3D-space. The semi-axes *a* and *c* are aligned along each symmetry axis, each indicating the equatorial radius in the *xy*-plane and the distance from the spheroid center to the pole along the symmetry axis of z, where *a*>*c* forms an oblate spheroid, while *a*<*c* a prolate. All coordinates of DDM 2O4 atoms in a micelle were utilized to describe the spheroidal shape of the micelle, which then were subjected to a parametric fitting for obtaining the semi-axes a and c. We found that both DDM120 and DDM150 create oblate spheroidal shapes (i.e., *a*>*c*), from which we estimated the effective surface area by using $A_{oblate} = 2\pi a^2 + \pi c^2/e \cdot \ln[(1+e)/(1-e)]$ (the eccentricity, *e* is defined by $e = [1 - c^2/a^2]^{1/2}$) (Kruger, DM & Kamerlin, SCL **2017** *ACS Omega* 2, 4524-4530).

(b) As the number of DDM molecules increases from 120 to 150, the equatorial dimension accordingly increases from *a* = 29.5 Å to 32.9 Å,. The area per DDM at the micellar surface is larger in DDM120 providing room for each DDM molecule to relax fast in the micelles relative to that in DDM150 (**Fig. 4b**). The experimental values obtained from small-angle X-ray scattering (SAXS) are also shown for comparison (Oliver RC, Lipfert J, Fox DA, Lo RH, Doniach S, et al. **2013** *PLoS ONE* 8: e62488). $N_{A,DDM}$: the aggregation number of DDM.
